# Low Density Lipoproteins Amplify Cytokine-signaling in Chronic Lymphocytic Leukemia Cells

**DOI:** 10.1016/j.ebiom.2016.11.033

**Published:** 2016-11-30

**Authors:** Lindsay McCaw, Yonghong Shi, Guizhi Wang, You-Jun Li, David E. Spaner

**Affiliations:** a Biology Platform, Sunnybrook Research Institute, Toronto, ON M4N 3M5, Canada; b Department of Immunology, University of Toronto, Toronto, ON M5S 1A8, Canada; c Department of Nutritional Sciences, University of Toronto, Toronto, ON, Canada; d Department of Medicine, University of Toronto, Toronto, ON M5G 2C4, Canada; e Department of Medical Biophysics, University of Toronto, Toronto, ON M5G 2M9, Canada; f Sunnybrook Odette Cancer Center, Toronto, ON M4N 3M5, Canada; g Department of Human Anatomy, College of Basic Medical Sciences, Jilin University, Changchun, Jilin 130021, China

**Keywords:** Chronic lymphocytic leukemia, Janus kinases, STAT3, Ruxolitinib, Cholesterol, Lipoproteins, Lysosomal lipase, HMGCR, Nuclear receptors

## Abstract

Recent studies suggest there is a high incidence of elevated low-density lipoprotein (LDL) levels in Chronic Lymphocytic Leukemia (CLL) patients and a survival benefit from cholesterol-lowering statin drugs. The mechanisms of these observations and the kinds of patients they apply to are unclear. Using an *in vitro* model of the pseudofollicles where CLL cells originate, LDLs were found to increase plasma membrane cholesterol, signaling molecules such as tyrosine-phosphorylated STAT3, and activated CLL cell numbers. The signaling effects of LDLs were not seen in normal lymphocytes or glycolytic lymphoma cell-lines but were restored by transduction with the nuclear receptor PPARδ, which mediates metabolic activity in CLL cells. Breakdown of LDLs in lysosomes was required for the amplification effect, which correlated with down-regulation of *HMGCR* expression and long lymphocyte doubling times (LDTs) of 53.6 ± 10.4 months. Cholesterol content of circulating CLL cells correlated directly with blood LDL levels in a subgroup of patients. These observations suggest LDLs may enhance proliferative responses of CLL cells to inflammatory signals. Prospective clinical trials are needed to confirm the therapeutic potential of lowering LDL concentrations in CLL, particularly in patients with indolent disease in the “watch-and-wait” phase of management.

## Highlights

• Slow-growing CLL cells use lysosomal lipase to break low density lipoproteins (LDLs) into free fatty acids and cholesterol.
• LdL degradation products increase survival of proliferating CLL cells.
• LDLs decrease oxidative stress and increase plasma membrane cholesterol.
• LDLs amplify signaling responses to cytokines but not antigens in proliferating CLL cells.
• Rapidly growing CLL cells, acute leukemia cells, and normal lymphocytes do not exhibit this dependence on LDLs.

## Graphical Abstract

**Figure:**
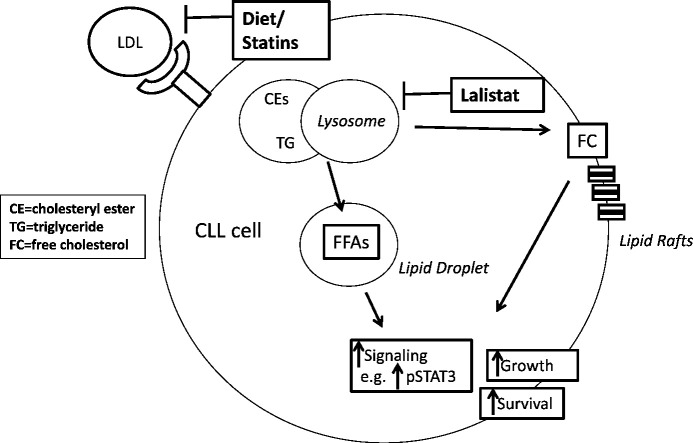

## 1 Introduction

Initiating and promoting events are required for the development of cancer. Initiation involves genetic lesions that transform normal cells while promoters stimulate transformed cells to proliferate and acquire more DNA changes to cause increasingly malignant behavior (Foulds, 1954). There is much interest in the idea that obesity and hyperlipidemia are tumor promoters (Goodwin and Stambolic, 2015).

Obesity is associated with a higher risk of developing many cancers, perhaps from increased levels of inflammatory cytokines and bioactive lipids that stimulate proliferation of cancer cells. Obesity is also a risk factor for dyslipidemias such as hypercholesterolemia. Low-density lipoproteins (LDLs) are the major carriers of cholesterol and have been studied mainly as cardiovascular risk factors but are increasingly recognized to play a role in cancer. LDLs promote proliferation, survival and migration of breast cancer cells (Nelson et al., 2013; dos Santos et al., 2014; Kitahara et al., 2011) and high LDL levels are associated with increased risk of prostate (Moses et al., 2009) and colorectal cancer (Holtzman et al., 1987). Statins lower LDL levels by blocking 3-hydroxy-3-methyl-glutaryl-CoA reductase (HMGCR), the rate-limiting enzyme of cholesterol synthesis, and have anti-cancer properties (Mucci and Stampfer, 2014; Chae et al., 2014; Gronich and Rennert, 2013). Explanations for these observations are unclear.

Chronic lymphocytic leukemia (CLL) is the most common leukemia in the developed world. It is a heterogeneous disease with some patients never needing treatment in their life-time while others exhibit a rapidly progressive course that can be fatal (Fabbri and Dalla-Favera, 2016). We found that LDL levels are elevated in up to 75% of CLL patients attending a specialized clinic at a single center and that statins delayed the need for chemotherapy in these patients by nearly 3 years (Chow et al., 2016) We also recently used administrative databases in a population-based case-control study involving 2124 CLL patients and 7935 controls to demonstrate a significantly higher incidence of hypercholesterolemia before a diagnosis of CLL and a survival benefit of 3.7 years for patients taking statins (Mozessohn et al., 2016).

Progression of CLL takes place in lymphoid organ microenvironments called pseudofollicles where leukemic cells are stimulated to proliferate by signals from B cell receptor (BCR) and toll-like receptor (TLR) ligands, TNF-family members, cytokines, and chemokines (Li et al., 2015, Herishanu et al., 2011). CLL cells with aggressive clinical behavior exhibit greater proliferative responses to microenvironmental signals (Tomic et al., 2011). Circulating CLL cells are readily obtained but studies of pseudofollicles require the use of *in vitro* models. We have found that much of the biology of pseudofollicles is captured by culturing circulating CLL cells with IL2, to represent T cell activity, along with the TLR7-agonist Resiquimod (Oppermann et al., 2016). The studies in this manuscript were designed to try to understand why hypercholesterolemia is apparently a tumor promoter for CLL by using this *in vitro* pseudofollicle model to observe how LDLs affect the biology of proliferating CLL cells.

## 2 Materials and Methods

### 2.1 Antibodies and Reagents

Fluorescent human CD19 and CD5 antibodies were from Pharmingen (San Francisco, CA). IL10-receptor (CD210) antibodies were from eBioscience (San Diego, CA) while IL10, IL10-receptor blocking antibodies, and low-density lipoprotein receptor antibodies were from R&D Systems, Inc. (Minneapolis, MN). The IL6-receptor blocking antibody Actemra (Roche Canada, Mississauga, ON), IL2 (Chiron, Corp., San Francisco, CA), and IFNα2b (Schering Canada Inc., Pointe-Claire, QC) were purchased from the Sunnybrook Cancer Centre pharmacy. 7-aminoactinomycin D (7-AAD) was obtained from Biolegend (San Diego, CA). Fatty acid–free bovine serum albumin, Nile Red, α-tocopherol, methyl-β-cyclodextrin, resiquimod, water-soluble cholesterol, oleic acid, phorbol dibutyrate (PDB), and β-actin antibodies were from Sigma-Aldrich (St. Louis, MO). Phospho-(Y705) STAT3 (Cat. No 9131), total STAT3, phospho-p44/42 MAPK(Erk1/2)(Thr202/Tyr204) (Cat. No 9102), phospho-(Ser17) SRC (Cat. No 5473), phospho-(Thr308)AKT (Cat. No 9275), and secondary horseradish peroxidase–conjugated anti-rabbit and anti-mouse antibodies (Cat. Nos. 7074 and 7076, respectively) were from Cell Signaling Technology (Beverly, MA). Low-, high-and very low-density lipoproteins were from EMD Chemicals (San Diego, CA). Lalistat (Hamilton et al., 2012) was a generous gift from Synageva BioPharma (Lexington, MA, USA). Perfringolysin O (PFO), a cytolysin from *Clostridium perfringens* that binds cholesterol in target membranes, was a gift from Alejandro Heuck (University of Massachusetts, Amherst, MA, USA). Ruxolitinib and Ibrutinib were from SelleckChem (Houston, TX, USA). Goat anti-human IgM Fc-specific antibodies were from Jackson ImmunoResearch Labs (West Grove, PA, USA). The Amplex® Red Assay Kit was from Invitrogen™. RPMI 1640 cell culture media was from Wisent Bioproducts (Quebec, Canada). The chemically defined CD lipid extract was from Thermo-Fisher Scientific (Mississauga, ON, Canada).

### 2.2 Purification of CLL Cells and Normal Lymphocytes

CLL cells were isolated as before by negative selection from the blood of consenting patients (Tomic et al., 2011), diagnosed with CLL by a persistent monoclonal expansion of CD19^+^ CD5^+^ IgM^lo^ lymphocytes. The cells were used directly for experiments. Patients had not been treated for CLL for at least 6 months prior to blood collection. Peripheral blood mononuclear cells (PBMCs) were isolated by centrifugation over ficoll gradients as before (Spaner et al., 2006). Protocols were approved by the Sunnybrook Research Ethics Board (PIN 222-2014).

### 2.3 Cell Culture

Unless specified otherwise, purified CLL cells and PBMCs were cultured at 1 × 10^6^ cells/ml in RPMI-1640 medium supplemented with transferrin and 0.25% fatty-acid free albumin in 6-, 12-or 24-well plates (BD Labware) at 37 °C in 5% CO_2_ for the times indicated in the figure legends.

### 2.4 Flow Cytometry

Viable cells were indicated by 7AAD-exclusion and reactive oxygen species (ROS) by 2′7′-dichlorofluorescin diacetate (DCFH2-DA; Molecular Probes) as before (Tomic et al., 2011; Tung et al., 2013). Nile Red and PFO were used to indicate lipoprotein-uptake by activated CLL cells. Nile Red reflects lipid droplets (Listenberger and Brown, 2007) that form in the presence of increased intracellular fatty acids and cholesterol. PFO was conjugated to Alexa Fluro 488 fluorochrome and used to measure plasma membrane cholesterol (Johnson et al., 2012). One million cells were stained with 3 μl 7AAD for 10 min, 3 μM Nile Red for 20 min, or 5 μl PFO for 15 min at room temperature or with 10 μM DCFH2 for 30 min at 37 °C. Cells were then washed in PBS and 10,000 viable events collected with a FACScan flow cytometer using Cellquest software (Becton Dickinson). Data was analyzed using FLOWJO software (Ashland, OR, USA). DCFH2 oxidation was measured as “green” (FL1) fluorescence on a log-scale.

### 2.5 PPARD^hi^ Daudi Cells

Human *PPARD* full-length cDNA was obtained from Addgene (Cambridge, MA, USA) and sub-cloned into the *Xho*I and *Eco*RI sites of retroviral MSCV2.2 plasmids or into the *Xho*I and *Not*I sites of lentiviral pLemiR plasmids. Construct sequences were confirmed before transfection. Replication-defective viruses were made by transfecting the MSCV-PPARD viral plasmid into the helper-free packaging cell line GP + A (B8), as described before (Wang et al., 2016). CD5^+^-Daudi cells (Spaner et al., 2013) at 2 × 10^6^ cells/ml were infected with supernatants from the virus-producing cells. Stably transfected clones were obtained by limiting dilution and selection in G418 (Multicell). Transfection was conducted with Lipofectamine 3000 according to the manufacturer's protocol (Invitrogen, Carlsbad, CA, USA). Cells infected with retroviruses containing the empty vectors but otherwise handled in the same way were used as controls.

### 2.6 Immunoblotting

Protein extraction and immunoblotting were performed as before (Tomic et al., 2011). Proteins were resolved in 10% sodium dodecyl sulfate-polyacrylamide gel electrophoresis and transferred to Immobilon-P transfer membranes (Millipore Corp., Billerica, MA). Western blot analysis was performed according to the manufacturers' protocol for each antibody. Chemiluminescent signals were created with SupersignalWest Pico Luminal Enhancer and Stable Peroxide Solution (Pierce, Rockford, IL) and detected with a Syngene InGenius system (Syngene, Cambridge, United Kingdom). For additional signal, blots were stripped for 60 min at 37 °C in Restore Western Blot stripping buffer (Pierce), washed twice in Tris-buffered saline plus 0.05% Tween-20 at room temperature, and reprobed as required. Densitometry was performed using Image J software. The densitometry value for each sample was normalized against the value for β-actin to obtain the intensities for phosphorylated-STAT3, -ERK, -AKT or -SRC reported in the figures.

### 2.7 Sample Preparation for Amplex® Red Assay Kit

The detergent/surfactant-free lysis/extraction method recommended by the manufacturer was used to remove chemical or cellular components that might interfere with the activity of the enzymes or dye. CLL cells (1 × 10^6^ cells) were purified, homogenized immediately in 200 μl chloroform-methanol (2:1 ratio), and centrifuged for 10 min at 14,000 rpm at room temperature in a microcentrifuge. The organic phase was collected and vacuum dried for 20 min using a DNA Speed Vac (DNA 110) from Savant. Dried lipids were dissolved in 200 μl of a 1 × concentration of reaction buffer contained in the Amplex® Red Assay Kit.

### 2.8 Amplex® Red Assay

Cholesterol samples (50 μl), prepared as above, were added to 96 well flat-bottom plates (BD Labware) along with 150 μM of the Amplex® Red reagent and incubated for 30 min at 37 °C. Fluorescence was then measured in a microplate reader with excitation at 540 nm and emission at 590 nm. Cholesteryl esters are hydrolyzed to cholesterol by cholesterol esterase in the reaction mix and then oxidized by cholesterol oxidase to yield hydrogen peroxide and a ketone product. Amplex Red reagent is a colorless substrate that reacts with hydrogen peroxide to produce resorufin, which has a fluorescence emission of around 585 nm.

### 2.9 Real-time PCR

RNA was prepared with the RNeasy mini kit (Qiagen, Valencia, CA, USA), and cDNA was synthesized from 2 μg of RNA using Superscript III reverse transcriptase (Life technologies, Invitrogen), according to the manufacturer's instructions. IL-10, HMGCoA reductase and hypoxanthineguanine phosphoribosyl transferase (HPRT) transcripts were amplified with the following primers: IL-10 forward, CATCGATTTCTTCCCTGTGA; reverse, CGTATCTTCATTGTCATGTAGGC; HMGCoA reductase forward, TGCATTAGACCGCTGCTATTC; reverse, GAATAGCAGCGGTCTAATGCA; HPRT forward, GAGGATTTGGAAAGGGTGTT; reverse, ACAATAGCTCTTCAGTCTGA. Polymerase chain reactions were carried out in a DNA engine Opticon System (MJ Research, Waltham, MA, USA) and cycled 34 times after initial denaturation (95 °C, 15 min) with the following parameters: denaturation at 94 °C for 20 s; annealing of primers at 58 °C for 20 s, and extension at 72 °C for 20 s. Abundance of mRNA transcripts was evaluated by a standard amplification curve relating initial copy number to cycle number. Copy numbers were determined from two independent cDNA preparations for each sample. The final result was expressed as the relative fold change of the target gene to HPRT.

### 2.10 Cytokine Measurements

A kit for human interleukin-10 (IL-10) was used according to the manufacturer's instructions (eBioscience). Concentrations were determined from standard curves. The assay was linear between 30 and 1000 pg/ml of IL10.

### 2.11 Cholesterol Manipulations

Methyl-β-cyclodextrin is a water-soluble structure that forms soluble inclusion complexes with cholesterol (Zidovetzki and Levitan, 2007). To load cells with cholesterol, water-soluble cholesterol (Sigma-Aldrich) packaged inside a methyl-β-cyclodextrin ring structure was added to purified CLL cells at a concentration of 15 μM. To strip cholesterol from cell membranes, empty methyl-β-cyclodextrin (Sigma-Aldrich) was added at a concentration of 0.5 mM.

### 2.12 Statistical Analysis

Paired *t-*tests with parametrics were used to determine p values with values < 0.05 considered significant. Pearson correlation statistical methods were used for the correlation analysis. A modified binary search algorithm (Knuth, 1971) was used to iteratively partition data in two dimensions.

### 2.13 Role of the Funding Source

The agencies that supported this work (LLSC and CIHR) had no role in the study design, data analysis, or manuscript preparation and submission.

## 3 Results

### 3.1 LDLs Increase Cellular Lipids, CLL Cells, and Signal Transduction *In Vitro*

**Fig. 1:**
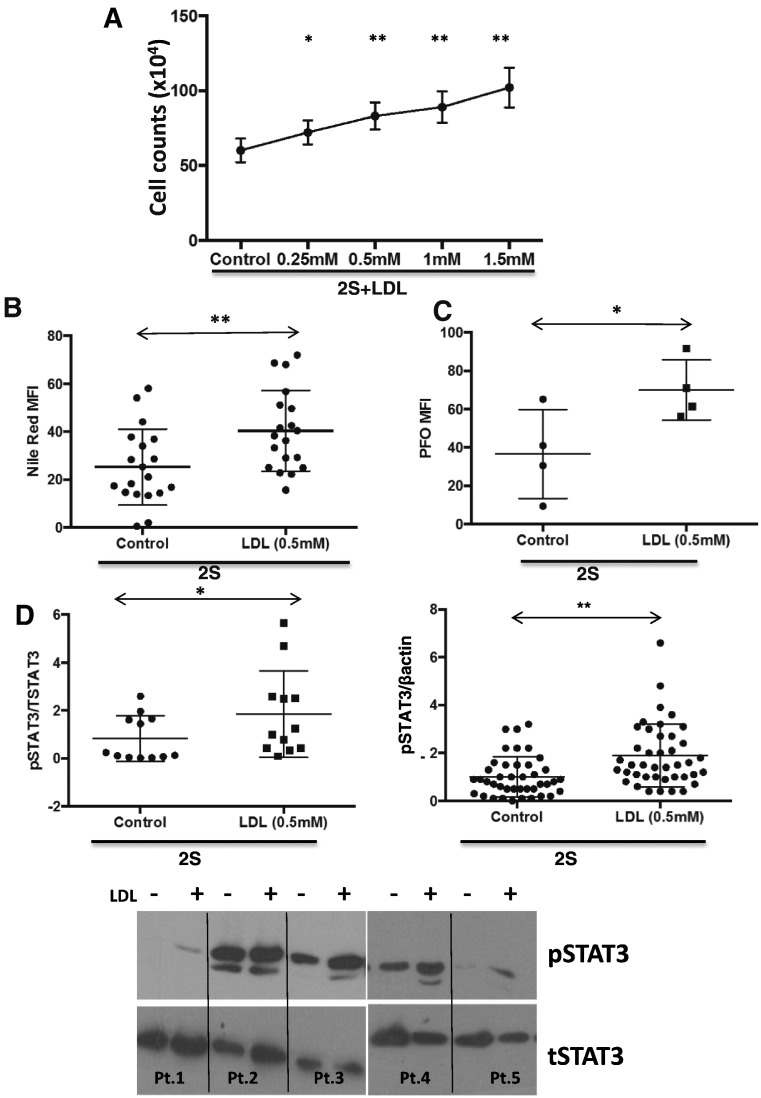
Effect of LDLs on growth, cellular lipids, and phosphorylated STAT3 levels in activated CLL cells *in vitro*. A. Purified CLL cells (1 × 10^6^ cells/ml) were stimulated with IL-2 and Resiquimod (2S) in lipid-poor media in the indicated concentrations of LDLs. Viable cells were counted manually by Tryphan Blue exclusion after 4 days. The average and standard deviation of the results from 6 different patient samples are shown. B,C. Mean fluorescence intensities (MFIs) of Nile Red staining (n = 19) to indicate lipid droplets (B) and PFO staining (n = 4) to indicate plasma membrane cholesterol levels (C) were measured by flow cytometry after 18 h and 24 h, respectively, in the presence or absence of 0.5 mM LDLs. Averages and standard deviations are shown. D. Protein extracts were made after 18 h and phospho-STAT3 expression measured by immunoblotting and quantified by densitometry with β-actin (right panel) or total STAT3 (left panel) used as loading controls. Averages and standard deviations are indicated and representative examples of immunoblots are shown below the graphs. *p < 0.05; **p < 0.01.

CLL cells were purified from the blood of CLL patients and activated in lipid-poor conditions with IL2 and resiquimod to represent stimulatory signals in pseudofollicles (Oppermann et al., 2016). Addition of LDLs increased viable cell numbers, counted by trypan-blue exclusion, in a dose-dependent manner (Fig. 1A). Based on the dose response curve, 0.5 mM LDLs were used in most subsequent experiments. Nile Red-staining suggested LDLs were taken up and incorporated into the cells to increase lipid levels (Fig. 1B) and PFO-staining suggested plasma membrane cholesterol was increased by LDLs in CLL cells (Fig. 1C).

The effects of LDLs on signaling molecules such as ERK, AKT, and STAT3 that mediate growth and proliferation of CLL cells were then studied by immunoblotting following 18 h of activation (Li et al., 2015, Longo et al., 2008, Yaktapour et al., 2014). This time-point was chosen to allow sufficient time for LDLs to be metabolized by leukemia cells. Following incubation with LDLs, pSTAT3 (Fig. 1D) and pERK, pAKT, and pSRC (Supplementary Fig. 1) increased significantly in activated CLL cells. Note that LDLs did not amplify signaling in circulating CLL cells that were not otherwise activated (Supplementary Fig. 2).

### 3.2 The Effects of LDLs on CLL Cells Are Not Seen in Normal Lymphocytes or Leukemic Cell-lines

**Fig. 2:**
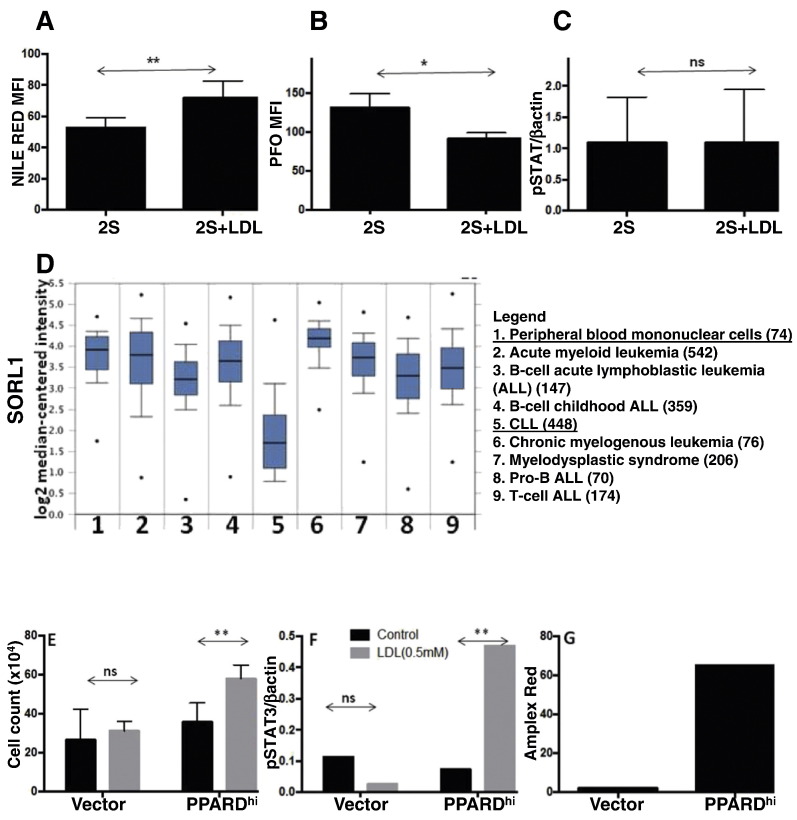
Effect of LDLs on normal lymphocytes and leukemic cell-lines. A–C. Purified normal PBMCs (1 × 10^6^ cells/ml) were stimulated with IL-2 and Resiquimod (2S) and cultured for 18 h with or without LDL (0.5 mM). Nile Red- (A) (n = 4) and PFO (B)-staining (n = 5) were measured by flow cytometry. C. Changes in pSTAT3 levels were determined by immunoblotting with total STAT3 protein levels used as a loading control (n = 5). D. Oncomine analysis of the Haferlach data base comparing 74 normal PBMC and 448 CLL samples indicates significantly lower *SORL1* expression in CLL cells compared to PBMCs and other hematologic malignancies. E–F. Vector control and PPARD^hi^ Daudi were cultured in RPMI-1640 ± LDL (0.5 mM). Cells were counted after 4 days (E) and pSTAT3 levels measured after 18 h (F). G. Cholesterol levels in control and PPARD^hi^ Daudi cells in exponential growth in RPMI + 5% FCS were measured by Amplex red staining. *p < 0.05; **p < 0.01; ns = not significant.

To determine if these effects were unique to CLL, a similar experimental approach was used to study PBMCs from normal donors (Fig. 2). We have shown previously that normal lymphocytes respond to IL2 and TLR-agonists more strongly than CLL cells in terms of cytokine production (Spaner et al., 2006). PBMCs took up LDLs, indicated by increased Nile red-staining (Fig. 2A), but did not increase plasma membrane cholesterol levels or change the magnitude of STAT3-phosphorylation in PBMCs treated with IL2 and resiquimod after 18 h (Fig. 2B, C).

These results suggested normal blood cells handled cholesterol from LDLs differently than CLL cells. To gain insight into a possible mechanism, the public Oncomine database (Rhodes et al., 2004) (http://www.oncomine.com/) was queried with the search terms: Concept: “cholesterol metabolism - GO Biological Process;” Analysis Type: Cancer *vs.* Normal Analysis; Cancer Type: Chronic Lymphocytic Leukemia. A number of genes involved in intracellular cholesterol transport were consistently down-regulated in CLL cells compared to normal PBMCs or B cells (Fig. 2D). In particular, *SORL1*, which controls cholesterol ester levels by inhibiting lipolysis from lipid droplets (Schmidt et al., 2016), was highly down-regulated in 6/7 independent gene arrays. Results with the Haferlach data set (Haferlach et al., 2010) are shown in Fig. 2D and suggest *SORL1* is expressed at lower levels in CLL cells than normal PBMCs as well as other hematologic cancers such as acute lymphoblastic and myeloid leukemia, chronic myeloid leukemia, and myelodysplastic syndrome (Fig. 2D).

Consistent with the possibility that cholesterol was handled differently by CLL cells than other blood cancers, growth and phospho-STAT3 levels in Daudi cells, a Burkitt's lymphoma model, were not increased when LDLs were added to serum-free media (Fig. 2E, F). However, the metabolic properties of Daudi cells are quite different from CLL cells (Spaner et al., 2013). Similar to acute leukemia cells, Daudi cells employ a metabolic strategy that is highly dependent on glycolysis. In contrast, CLL cells are more dependent on fatty acid oxidation due to their relatively high expression and/or activity of two members of the nuclear peroxisome proliferator activated receptor (PPAR) family PPARα (Spaner et al., 2013, Masoodi et al., 2014) and PPARδ (Supplementary Fig. 7). To make a cell-line with greater resemblance to CLL, Daudi cells were transduced with a *PPARD*-expression vector. In contrast to control cells infected with the expression vector alone, PPARD^hi^ cells exhibited the behavior of activated CLL cells as they increased in number (Fig. 2E) and expressed higher levels of pSTAT3 (Fig. 2F) in the presence of LDLs. Similar to activated CLL cells with added LDLs (Fig. 1C), cholesterol levels of exponentially growing PPARD^hi^ Daudi cells in serum-containing media were higher than vector control cells (Fig. 2G).

### 3.3 Increased STAT3 Phosphorylation Involves IL10 But Not Increased IL10 Production or Receptor Expression

**Fig. 3:**
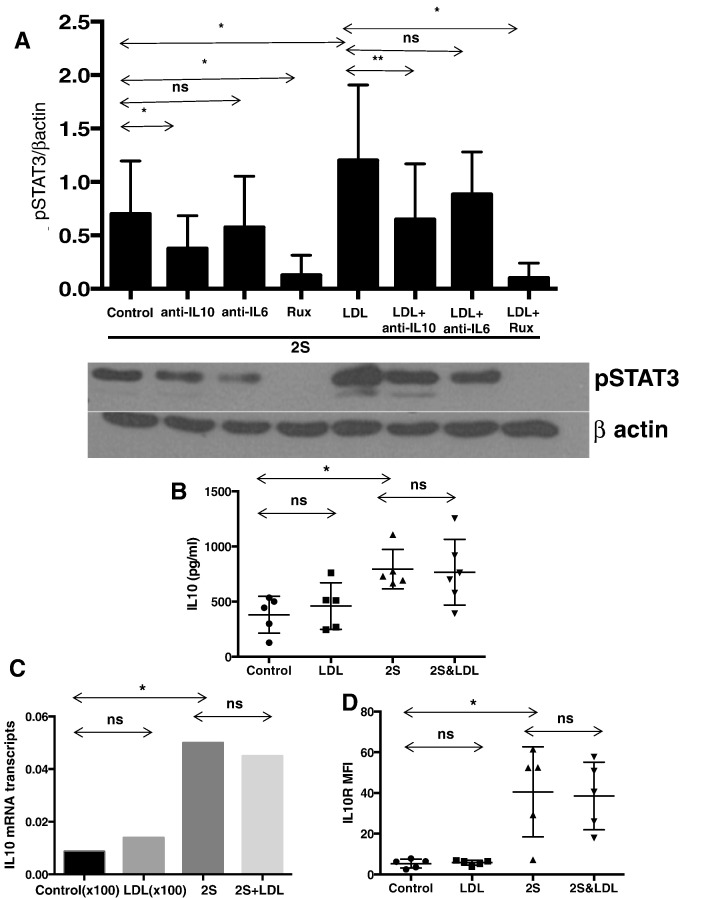
Effect of LDLs on cytokine-signaling in activated CLL cells *in vitro*. Purified CLL cells were cultured in lipid-poor conditions with or without IL-2 and Resiquimod and with or without addition of LDLs (0.5 mM) in the presence or absence of IL-6 antibodies (10 ng/ml), IL-10 antibodies (10 ng/ml) or Ruxolitinib (Rux) (500 nm). A. After 18 h, phospho-STAT3 levels were determined in 4 patient samples by immunoblotting and densitometry and normalized to the results for β-actin. The averages and standard deviations of the relative densitometric values are indicated in the graph and a representative immunoblot is shown. B, C, D. After 48 h, IL10 levels in the culture supernatants were measured by ELISAs (n = 5) (B), IL10 mRNA was measured by quantitative RT-PCR for Pt.117 (C), and mean fluorescence intensities of IL10-receptor (IL10R)-staining were determined by flow cytometry for 5 patient samples (D). Averages and standard deviations are shown. *p < 0.05; **p < 0.01; ns, non significant.

Studies to identify the mechanism of the increase in tyrosine-phosphorylated STAT3 levels (Fig. 1D) were undertaken due to the importance of this transcription factor in CLL biology (Tomic et al., 2011; Li et al., 2015; Spaner et al., 2016). STAT3 phosphorylation in response to TLR7- and IL2-signaling involves autocrine production of IL10 (Spaner et al., 2006). IL10-signaling in resting CLL cells is mediated by Janus kinases (JAKs) and can be blocked completely by the JAK-inhibitor Ruxolitinib (Li et al., 2015) and partially by IL10-blocking antibodies at 10 ng/ml in the presence or absence of LDLs (Supplementary Fig. 3). Both inhibitors prevented the LDL-mediated increase in phospho-STAT3 in CLL cells activated with IL2 and resiquimod (Fig. 3A). Anti-IL6 blocking antibodies also partially inhibited STAT3 phosphorylation (Fig. 3A). These findings suggested that signals transmitted through JAKs were enhanced by LDLs.

The observations might be explained by LDL-mediated increases in autocrine cytokine production or cytokine receptor levels. STAT3 is phosphorylated by a number of cytokines including IL6 and type 1 interferons (IFNs) following activation of CLL cells by IL2 and resiquimod (Tomic et al., 2011; Li et al., 2015; Spaner et al., 2016) but we focused first on IL10. Surprisingly, IL10 in culture supernatants measured by ELISAs after 48 h (Fig. 3B) and *IL10* mRNA levels (Fig. 3C) were not increased by LDLs. IL10-receptor (IL10R) levels measured by flow cytometry also did not change (Fig. 3D). One explanation for these observations was that signaling responses to IL10 had been potentiated by LDLs.

### 3.4 Lysosomal Degradation Is Required for LDLs to Increase STAT3-phosphorylation

**Fig. 4:**
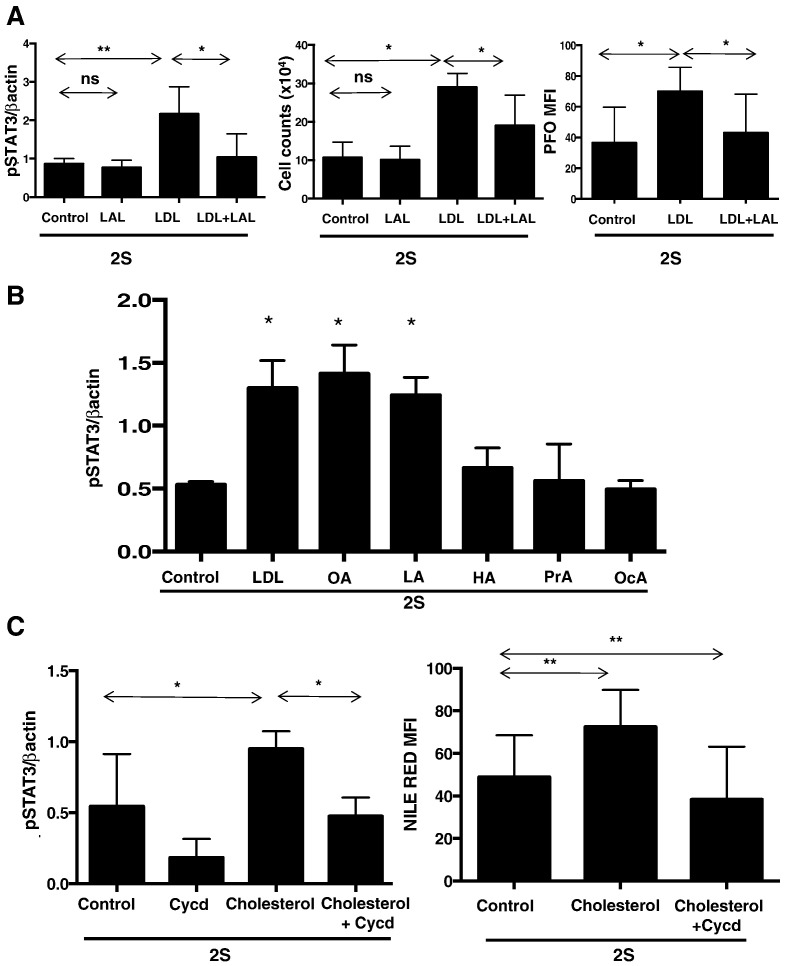
Effect of LDL components on STAT3-phosphorylation in activated CLL cells. A. CLL cells were activated with IL-2 and Resiqimod (labeled “2S”) in the presence and absence of LDLs (0.5 mM) and/or Lalistat (LAL) (1 μM). Densitometric values of p-STAT3 normalized to β-actin expression were determined after 18 h (left panel) (n = 5), cell counts were measured after 4 days by trypan blue exclusion (middle panel) (n = 3), and mean fluorescence intensities of PFO staining to indicate plasma membrane cholesterol levels were measured by flow cytometry after 24 h (right panel) (n = 4). B. 2S-activated CLL cells from 3 different patients were cultured with or without LDL (0.5 mM), oleic acid (OA) (5 μm), linoleic acid (LA) (5 μm), heptanoic acid (HA) (5 μm), propanoic acid (PrA) (5 μm), or octanoic acid (OcA) (5 μm) and normalized phospho-STAT3 levels determined after 18 h. C. 2S-activated CLL cells from 20 different patients were cultured with or without cholesterol (15 μm) and/or methyl-β-cyclodextrin (Cycd) (0.5 mM). Expression of pSTAT3 normalized to β-actin (left panel) was determined for each sample after 18 h and Nile Red-staining measured by flow cytometry to confirm lipid-loading and stripping (right panel). Averages and standard deviations are shown in each graph. *p < 0.05; **p < 0.01; ns, non significant.

Following uptake into cells, LDLs are degraded in lysosomes into cholesterol, free fatty acids, and other components. Cholesterol esters are metabolized by lysosomal lipase, which can be inhibited by the small molecule Lalistat (Hamilton et al., 2012). Lalistat prevented the increases in STAT3-phosphorylation and cell numbers caused by LDLs (Fig. 4A, left and middle panels). PFO-staining increased following incubation with LDLs, consistent with delivery of more cholesterol to plasma membranes (Johnson et al., 2012), and was decreased by Lalistat, consistent with inhibited breakdown of LDLs to free cholesterol (Fig. 4A, right panel). These observations suggested that products of cholesterol esters and perhaps other molecules in LDLs were responsible for increased growth and signaling in activated CLL cells.

### 3.5 Free Fatty Acids, Cholesterol, and Vitamin E Increase Phospho-STAT3 in CLL Cells

LDLs deliver free cholesterol, free fatty acids, and vitamin E to cells. These components were studied individually in CLL cells activated in lipid-poor conditions. Oleic and linoleic acid, which are long chain fatty acids carried by cholesterol esters in LDLs, significantly increased STAT3 phosphorylation in CLL cells (Fig. 4B). In contrast, phospho-STAT3 levels were not increased by the short-chain free fatty acid propionic acid and medium-chain fatty acids heptanoic and octanoic acid, which are not transported by LDLs (Fig. 4B).

Free cholesterol, solubilized in methyl-β-cyclodextrin, also increased STAT3 phosphorylation in activated CLL cells, which was reduced by removing cholesterol with empty methyl-β-cyclodextrin (Fig. 4C, left panel). Cellular uptake of cholesterol was confirmed by Nile Red-staining (Fig. 4C, right panel).

**Fig. 5:**
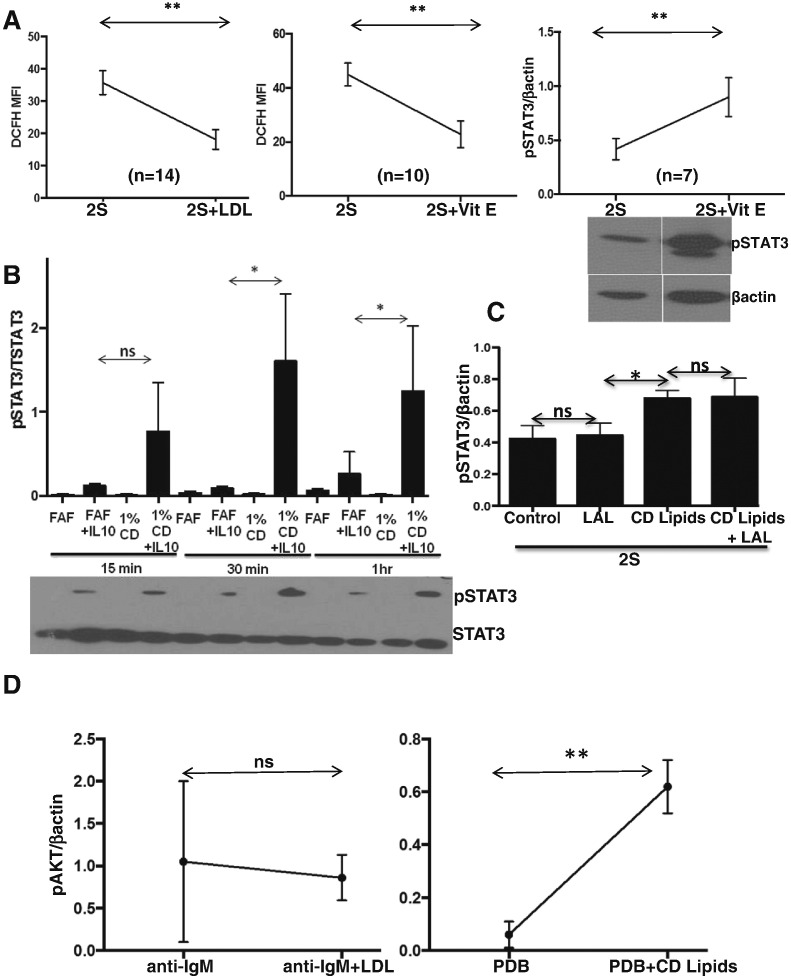
Effect of LDL components on oxidative stress and signaling in CLL cells. A. CLL cells were activated with IL2 and resiquimod (2S) and with or without LDL (0.5 mM) or Vitamin E (5 μM). Mean fluorescence intensities (MFIs) of DCFH staining were determined by flow cytometry after 12 h (left and middle panels) and densitometric values of p-STAT3 expression relative to β-actin were determined after 18 h (right panel). Shown are the average results and standard errors from the number of patient samples indicated in each graph. An example of an immunoblot is shown in the insert. B. Purified CLL cells (1 × 10^6^ cells/ml) were cultured over-night in fatty acid free media (FAF) or 1% CD Lipid extract, consisting of a mixture of free cholesterol, fatty acids, and Vitamin E. The cells were then treated with IL10 (10 ng/ml) but not otherwise activated with IL2 and resiquimod. Levels of pSTAT3 were measured by immunoblotting after 15 min, 30 min, and 1 h using STAT3 as the loading control. Averages and standard deviations of relative pSTAT3 densitometry values for 6 different patient samples are plotted with a representative immunoblot shown below the graph. C. Relative pSTAT3 values were measured in CLL cells from 6 different patients that had been activated with IL2 and resiquimod for 18 h in fatty acid free media (FAF) or 1% CD Lipid extract with or without Lalistat (LAL) (1 μM). Averages and standard deviations are shown. D. CLL cells were cultured with or without LDL (0.5 mM) for 18 h and stimulated with anti-IgM antibodies (10 ng/ml) (n = 3; left panel) or phorbol dibutyrate (5 ng/ml) (n = 3; right panel). Phospho-AKT (pAKT) levels were determined after 30 min by immunoblotting with β-actin as a loading control. *p < 0.05; **p < 0.01; ns, non significant.

Reactive oxygen species (ROS) can also alter STAT3-phosphorylation by affecting JAK/STAT signaling (Tomic et al., 2011) and the antioxidant vitamin E is carried with cholesterol esters in LDLs (Bowry et al., 1992). Both LDLs and vitamin E decreased ROS levels in activated CLL cells, as measured by DCFH-staining (Fig. 5A, left and middle panels) (Tomic et al., 2011). Vitamin E also increased phospho-STAT3 in activated CLL cells (Fig. 5A, right panel).

### 3.6 Fatty Acids, Cholesterol and Vitamin E Increase IL10-signaling Responses in CLL Cells

The results of Fig. 4, Fig. 5A suggested LDLs deliver free fatty acids, cholesterol, and vitamin E to activated CLL cells and these lipid species can all affect the outcome of IL10-signaling. A mixture of free cholesterol, fatty acids, and vitamin E that would result from lysosomal degradation of LDLs should then phenocopy the effect of LDLs on cytokine-signaling. To test this idea, CLL cells were exposed to a chemically defined lipid extract (CD lipids) containing similar components as degraded LDLs and then activated directly with IL10. This design avoids the 18 h time-point that identified the amplification of signaling processes by LDLs (Fig. 1) but also allowed a cascade of other events such as phosphatase induction that could affect signaling. The lipid extract enhanced IL10-induced STAT3-phosphorylation (Fig. 5B) in the same way that LDLs enhanced STAT3 signaling in response to autocrine production of IL10 following stimulation with IL2 and resiquimod (Fig. 3). The lipid extract also increased pSTAT3-levels induced by IL2 and resiquimod despite the presence of Lalistat (Fig. 5C), as expected if degradation products of LDLs were responsible for this effect.

### 3.7 Signaling Pathways in CLL Cells Are Not Affected Universally By LDLs

Pathways other than IL10-signaling are likely to be affected if LDLs amplify signal transduction by increasing lipid content in CLL cells. To determine if signaling from other cytokines was affected by LDLs or LDL components, CLL cells were exposed to the CD lipid extract and activated with the type 1 inteferon IFNα2B (Tomic et al., 2011). Analogous to the results for IL10, IFN-induced STAT3-phosphorylation was also enhanced by the lipid extract (Supplementary Fig. 4).

Phorbol esters activate protein kinase C (PKC) signaling in CLL cells (Spaner et al., 2006), which can be indicated by changes in intracellular levels of phospho-AKT^T308^. Similar to the effects on IL10 and IFNα-signaling, phorbol dibutyrate (PDB) induced higher pAKT levels in CLL cells with higher lipid levels due to pre-incubation with the CD lipid extract (Fig. 5D, right panel).

To assess BCR-signaling, pAKT levels were measured as they usually increase following BCR-crosslinking (Longo et al., 2008). In contrast to the stronger effects on cytokine and PDB-signaling, LDLs did not significantly change AKT-phosphorylation (Fig. 5D, left panel) or subsequent proliferation (Supplementary Fig. 6) in response to Ig-crosslinking. Taken together, these results suggest that LDLs affect a subset of signaling pathways in CLL cells, especially those that transmit cytokine signals.

### 3.8 LDL-induced STAT3-phosphorylation Is Associated With Down-regulation of *HMGCR*, Low Numbers of Circulating Leukemic Cells, and Slow Doubling Times *In Vivo*

**Fig. 6:**
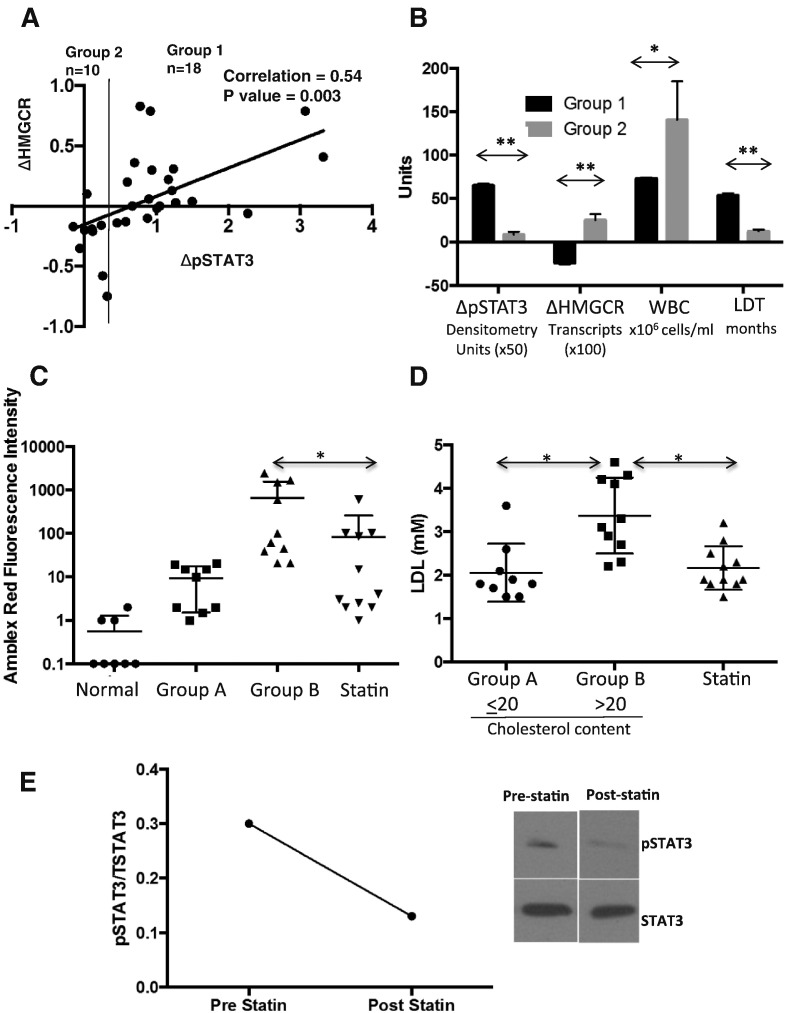
Effect of LDLs on STAT3-phosphorylation and *HMGCR* mRNA expression *in vitro* and cholesterol content of CLL cells *in vivo*. A. CLL cells from individual patients were activated in lipid-poor media with IL2 and resiquimod with or without 0.5 mM LDLs. After 18 h, pSTAT3 levels were quantified by densitometry and normalized to the results for β-actin. *HMGCR* transcripts were measured by quantitative RT-PCR. The differences between the LDL-treated cells compared to the untreated cells (ΔpSTAT3 and ΔHMGCR) were calculated and their relationship explored with a scatter plot. Note that the negative value of ΔHMGCR is shown on the y-axis to reflect LDL-uptake. B. A cutoff of ΔpSTAT3 = 0.6 was used to empirically classify patients into group 1 (ΔpSTAT3 ≥ 0.6; n = 18) and group 2 (ΔpSTAT3 < 0.6; n = 11). Highly significant differences were seen in ΔHMGCR, white blood cell (WBC) numbers, and lymphocyte doubling times (LDTs) between the two groups. C, D. Cholesterol content of CLL cells from 30 other patients and normal B cells from 8 healthy donors were measured by Amplex-red fluorescence (C). LDL concentrations at the time of cell collection were taken from the patients' electronic medical records (D). The results are shown as 4 groups: normals, patients on statins, and CLL cholesterol concentrations higher or lower than 20 fluorescence units. Average results for each group were used for statistical analysis. E. Protein extracts were frozen immediately from CLL cells purified from the blood of a patient before and 3.5 months after starting Simavastin (20 mg daily). LDL concentrations pre- and post-statin therapy were 2.82 and 1.76 mM. Levels of pSTAT3 in the thawed extracts were determined at the same time using STAT3 as the loading control. The immunoblot is shown on the right with densitometry readings on the left. **p < 0.01; *p < 0.05.

While these results suggested a model whereby LDLs are degraded in lysosomes into free fatty acids, cholesterol, and vitamin E that enhance signal transduction in CLL cells by affecting lipid structures and cellular oxidative state, a number of samples did not exhibit enhanced STAT3-phosphorylation in the presence of LDLs (Fig. 1D, right panel). To gain more insight into this variability, CLL cells from 29 additional patients were activated in the presence and absence of LDLs. Phospho-STAT3 and *HMGCR* transcripts were then measured after 18 h. *HMGCR* levels correlate with internal cholesterol levels and can serve to indicate uptake of lipoproteins (Nohturfft and Zhang, 2009; Brown and Goldstein, 1980). Down-regulation of *HMGCR* reflects increased intracellular cholesterol stores, particularly in the endoplasmic reticulum. A direct correlation was observed between increased pSTAT3 phosphorylation and negative changes in *HMGCR* expression in most samples (Fig. 6A) as expected if LDLs were taken up and enhanced signaling.

These results also revealed that a group of samples did not up-regulate pSTAT3 expression and either did not down-regulate *HMGCR* expression or paradoxically increased it following treatment with LDLs (Fig. 6A). Failure to down-regulate *HMGCR* levels could reflect failure to internalize LDLs but flow cytometric measurements of LDL receptor expression and Nile Red-staining following exposure to LDLs were not consistent with this possibility (not shown). The clinical characteristics of this group of samples were not significantly associated with biological prognostic markers such as 17p or 11q deletions, CD38 expression, or serum β2M levels (Table 1). Note that Ig mutation status was not available for these samples. However, preliminary inspection of the clinical characteristics did suggest a correlation with lymphocyte doubling time (LDT). Using an unsupervised binary partitioning algorithm, we stratified the cohort based on the ΔpSTAT3-value that yielded the greatest statistical difference in LDT *i.e.* the samples were classed according to whether their relative densitometry values for pSTAT3 increased by more (Group 1) or less (Group 2) than 0.6 (Fig. 6A). The clinical characteristics of the patients are described in Supplementary Table 1 and summarized in Table 1 (top rows). Group 2 patients (whose CLL cells did not increase pSTAT3 expression and displayed altered cholesterol metabolism in that they failed to down-regulate *HMGCR*) had significantly higher WBC counts and much shorter lymphocyte doubling times, which are associated with more aggressive disease (Molica and Alberti, 1987) (Fig. 6B). Note that 8/11 group 2 patients went on to require treatment with an average time-to-treatment of 8.9 ± 0.99 months, including one patient who had a liver transplant with a clinical response to the anti-rejection drugs. In contrast, only 4/18 of the patients in group 1 required some form of treatment in the time-frame of this study, including one who received glucocorticoids to treat autoimmune hemolytic anemia.

### 3.9 Statin Use Is Associated With Lower Cholesterol in CLL Cells, Lower Circulating CLL Cell Numbers, and Longer Doubling Times *In Vivo*

If CLL cells respond to increased LDL levels by increasing their cholesterol content as suggested by down-regulated *HMGCR* expression, then a relationship between circulating LDLs and cholesterol content of CLL cells should exist *in vivo*. To address this possibility, circulating CLL cells were purified from an additional cohort of 30 patients described in Supplementary Table 2 and summarized in Table 1 (bottom rows), including 11 on statins for at least 6 months to lower blood cholesterol. Cholesterol content of the cells was measured by the Amplex Red assay as described in the materials and methods. Total cholesterol levels were higher in CLL cells than B cells from 8 normal donors but exhibited a wide range in the patient samples (Fig. 6C). The normal donors were not age-matched to the CLL patients. Five donors ranged between 25 and 35 years old and only three were older than 60 years, which is the demographic group of CLL. However, the oldest of the normal donors had the lowest B cell cholesterol levels (Fig. 6C).

Preliminary inspection of the data for the 19 patients not on statins revealed a relationship with plasma LDL level at the time of collection of the CLL cells and suggested a possible direct relationship of tumor cell cholesterol with exogenous LDL levels. Using the partitioning algorithm, we then stratified this cohort based on the mean fluorescence intensity of Amplex-red staining that yielded the greatest statistical difference in LDL level *i.e.* the samples were classed according to whether the mean fluorescence intensity of Amplex-red staining was < 20 (Group A = 9 patients) or > 20 (Group B = 11 patients) (Fig. 6D). Group A patients tended to have higher numbers of circulating leukemia cells, although this did not reach statistical significance (Table 1).

If high levels of LDLs increase fatty acid and cholesterol stores and responses to growth promoting signals (Fig. 1), then lowering exogenous LDL levels with statins should decrease intracellular cholesterol and slow the growth of CLL cells *in vivo*. Consistent with this idea, patients taking statins had lower LDL levels and significantly lower cholesterol levels in their CLL cells compared to group B patients (Fig. 6C, D). Remarkably, these patients also had fewer circulating leukemia cells and longer lymphocyte doubling times than both groups of untreated CLL patients (Table 1, bottom rows).

Formal proof that lowering LDL levels with statins *in vivo* decreases intracellular cholesterol levels and alters signaling processes in CLL cells requires an interventional clinical trial. However, blood samples were able to be collected from 1 patient before and 3.5 months after starting Simvastatin (20 mg daily) for consistently elevated LDL levels. As shown in Fig. 6E, pSTAT3 levels were present in circulating CLL cells prior to commencing the statin when the LDL level was 2.82 mM but were much lower on the second occasion when the LDL level was 1.76 mM. However, no significant change in circulating CLL cell numbers occurred over this time.

## 4 Discussion

The results in this manuscript provide additional support for a role of hypercholesterolemia in the pathogenesis of CLL (Chow et al., 2016; Mozessohn et al., 2016) and a possible mechanism in some patients. Internalization and lysosomal degradation of LDLs to free fatty acids, cholesterol, and vitamin E enhance growth-promoting signaling processes in activated CLL cells (Figs. 1A, D, Fig. 3, Fig. 4, Fig. 5). This ability of LDLs to alter lipid levels and signaling processes in CLL cells appears to apply mainly to patients with indolent disease characterized by long lymphocyte doubling times (Fig. 6).

The 30 patients whose samples were used to measure cholesterol levels in CLL cells (Fig. 6C, D) included 11 who were previously prescribed statins and 9 with LDL levels higher than 2.5 mM, the upper limit of normal at the Sunnybrook clinical laboratory. Accordingly, 67% of these patients had evidence for increased LDLs, consistent with our previous findings of high rates of hypercholesterolemia in CLL patients (Chow et al., 2016; Mozessohn et al., 2016). The reason LDLs are increased in CLL patients is not clear. Excess calorie intake is likely in some patients but hypercholesterolemia could also be a paraneoplastic syndrome. CLL cells make high amounts of oleoylethanolamide (OEA), a bioactive lipid that causes adipocytes to release free fatty acids, which could then be packaged in the liver into very low density lipoproproteins (VLDLs) and metabolized subsequently to LDLs to account for high rates of hypercholesterolemia (Masoodi et al., 2014).

The growth promoting effects of LDLs on CLL cells appear to be multi-factorial. LDLs increased the activity of a number of signaling pathways in an *in vitro* model of the CLL microenvironment (Fig. 1D, Supplementary Fig. 1), as exemplified by IL10-dependent STAT3 signaling (Fig. 3). Evidence for enhanced STAT3-signaling was also provided by the increase in total STAT3 proteins seen in some patient samples (*e.g.* Pt.2, Fig. 1D) because *STAT3* is an auto-regulated gene (Yang et al., 2007). Note that LDL products did not increase STAT3 protein levels in the absence of concomitant cytokine-signaling (Fig. 5B; Supplementary Fig. 2). LDLs did not increase IL10 production or receptor expression by activated CLL cells (Fig. 3), suggesting signaling was sensitized through the IL10-receptor. The results with Lalistat suggest that lysosomal degradation is necessary and many LDL components contribute to this sensitization (Fig. 4). Long-chain free fatty acids, free cholesterol, and vitamin E, which would be released into CLL cells following degradation of LDLs in lysosomes, individually phenocopied the increase in phospho-STAT3 levels following LDL-loading (Fig. 4, Fig. 5A). A mixture of free fatty acids, cholesterol, and vitamin E enhanced signaling responses to IL10 and another STAT3-activating cytokine, IFNα2b (Supplementary Fig. 4) and to PKC-activating phorbol esters (Fig. 5D, right panel). However, not all signaling processes relevant to CLL progression appear to be amplified by the increased lipid-content caused by LDLs. For example, BCR-signaling indicated by AKT-phosphorylation (Fig. 5D, left panel) and proliferation (Supplementary Fig. 6) was not affected by LDLs.

We speculate LDLs augment signaling efficiency in CLL cells in a number of ways. Free cholesterol can alter the structure of lipid rafts in the plasma membrane, affecting the physical assembly of cytokine receptor complexes and location of inhibitory molecules such as phosphatases (Delos Santos et al., 2015). Free fatty acids and cholesterol can be stored in lipid droplets, which are sources of bioactive lipids that can amplify growth-factor signaling (Yue et al., 2014). Lipid-loading with fatty acids and cholesterol causes endoplasmic reticulum (ER) stress that can activate signaling processes in cancer cells (Garris and Pittet, 2015). Vitamin E can affect signaling by manipulating the cellular redox state (Duhe, 2013). By amplifying responses to growth promoting signals in the CLL microenvironment, elevated levels of LDLs might enhance proliferation and survival of CLL cells *in vivo* and promote tumor progression. Lowering LDL levels might then slow the growth of CLL cells and delay progression in many patients (Chow et al., 2016; Mozessohn et al., 2016).

These observations appear to be unique to CLL cells compared to acute leukemias and normal lymphocytes (Fig. 2). However, the observations may apply to other cancers that are associated with hypercholesterolemia and slowed by statins (Kitahara et al., 2011, Gronich and Rennert, 2013). Hypercholesterolemia is associated with elevated numbers of circulating normal B lymphocytes due to effects on stromal cells and chemokines rather than changes in intrinsic B cell signaling (Rafii and Nolan, 2010). While LDLs increased lipid stores, they did not change plasma membrane cholesterol or cytokine signaling in normal PBMCs activated by IL2 and resiquimod (Fig. 2). Accordingly, intracellular cholesterol may be handled differently by CLL cells than by normal cells and other leukemias but more experiments are needed to address the possibility that differential expression of molecules like SORL1 (Fig. 2D) and PPARδ (Supplementary Fig. 6) explain disease-specific patterns of cholesterol uptake, storage, and efflux (Schmidt et al., 2016, Skogsberg et al., 2003).

In contrast to CLL (Chow et al., 2016, Mozessohn et al., 2016), patients with acute leukemia are characterized by aberrantly low LDL levels and acute leukemia cells exhibit low levels of intracellular cholesterol compared to normal cells (Usman et al., 2016). Unique metabolic properties of CLL cells as exemplified by the expression patterns of *SORL1* (Fig. 2D) may explain these differences. Acute leukemia cells are generally dependent on glycolysis while CLL cells are more dependent on fatty acid oxidation, which is regulated by the PPAR nuclear receptor family (Spaner et al., 2013). Consistent with this idea, LDLs did not affect glycolytic Daudi cells significantly but increased intracellular cholesterol, growth, and STAT3-phosphorylation of Daudi cells that were transduced with *PPARD* to make them more “CLL-like” (Fig. 2C).

Handling of LDLs is also heterogenous in CLL cells. For example, signaling processes in CLL cells appear less dependent on exogenous LDLs in a group of patients with more aggressive disease, indicated by higher circulating leukemia cell numbers and rapid doubling times *in vivo* (Fig. 6A, B). An interpretation of these findings may be that relatively early-stage CLL cells with indolent growth are more dependent on LDLs. However, as they become more aggressive in terms of rate of proliferation, they change their metabolism to become more like mitogen-activated normal lymphocytes (Owens et al., 1990) or acute leukemia cells, *i.e.* they become less responsive to exogenous cholesterol (Fig. 2, Fig. 6A, B) and decrease their intracellular cholesterol levels (Fig. 6C, D). Multivariate analysis of our prior clinical study revealed that the protective effects of statins did not apply to patients with high-risk cytogenetic lesions associated with aggressive disease (Chow et al., 2016). However, we could not identify common cytogenetic abnormalities in group 2 patients (Table 1) and querying Oncomine (Rhodes et al., 2004) with the search terms: Gene: “HMGCR” and Cancer Type: Chronic Lymphocytic Leukemia did not reveal any correlation of aggressive prognostic markers (*i.e.* unmutated IGVH genes or 11q and 17 deletions) with *HMGCR* expression.

Taken together, the observations in this manuscript suggest there is a cohort of CLL patients whose leukemia cells internalize LDLs and amplify signaling properties and for whom LDLs are tumor promoters. By lowering LDL levels, statins may reduce signaling and slow the growth of these cells, explaining their ability to slow time to first treatment and increase overall survival. (Chow et al., 2016, Mozessohn et al., 2016) However, statins may also have direct anti-tumor effects that are independent of plasma LDL-concentrations (Chapman-Shimshoni et al., 2003). A clinical trial comparing dietary strategies to lower cholesterol (Jenkins et al., 2011) with statins, especially in patients with elevated baseline LDL-cholesterol levels and relatively indolent disease (Fig. 6), is needed to determine the relevance of the mechanism proposed here that statins exert their beneficial effects by lowering intracellular cholesterol, antioxidants, and growth promoting signal transduction in CLL cells (Figs. 5A, 6E). Appropriate therapeutic targets for LDL-cholesterol to prevent tumor progression are unknown, in contrast to target levels required to prevent cardiovascular disease (Stone and Lloyd-Jones, 2015). Moreover, the kinase inhibitor Ibrutinib lowers cytokine levels in CLL patients (Bachireddy and Wu, 2016) and prevents STAT3-phosphorylation in the pseudofollicle model regardless of LDLs (Supplementary Fig. 5). An arm of the trial should also compare kinase inhibitors that block tumor-promoting signaling directly with lowering LDL plasma levels to decrease signaling-strength in the watch-and-wait phase of CLL management.

The following are the supplementary data related to this article.

Table 1 Patient data. Table 1

Table 2 Patient data for CLL cholesterol and LDL analysis. Table 2

Supplementary figures Image 2

## Funding

CIHR and LLSC.

## Conflict of Interest

The authors declare no conflicts of interest with respect to this work.

## Author Contributions

LM and DS designed the research, analyzed the data, and wrote the paper. YS and GW helped design and perform experiments. Y-JL generated PPARD^hi^ Daudi cells.

## Figures and Tables

**Table 1:** Summary of patient data.

	Sex	Age (yrs)	Time (yrs)^a^	WBC (× 10^6^/ml)	Stage^b^	CD38 (%)	β2m^c^	FISH	Tx^d^	LDT (mos)^e^	Δp-STAT3^f^	ΔHMGR^g^	LDL (mM)	Chol (MFI)	Statin use
Group 1 (n = 18)	0.5^h^	67.7 ± 2.3^j^	9.6 ± 1.2	73 ± 4.9	2.7 ± 0.3	5.2 ± 1.7	3.6 ± 0.5	4/9^i^	0.8 ± 0.3	53.6 ± 10.4	1.3 ± 0.18	− 0.24 ± 0.07	2.38 ± 0.24	NA	11/18
Group 2 (n = 11)	0.45	65.7 ± 2.5	11.9 ± 2.8	140.6 ± 44.3	2.7 ± 0.4	7.7 ± 3.5	4.2 ± 1.0	3/7	0.8 ± 0.4	12.6 ± 1.7	0.17 ± 0.07	0.25 ± 0.07	2.47 ± 0.26	NA	6/11
				⁎						⁎⁎	⁎⁎	⁎⁎			
Group A (n = 9)	0.55	70.7 ± 5.2	10.6 ± 1.7	154.5 ± 40.9	3.4 ± 0.3	6.2 ± 2.7	5.0 ± 1.3	3/8	1.3 ± 0.4	29.3 ± 9.7			2.1 ± 0.2	9.5 ± 2.7	no
Group B (n = 10)	0.8	66 ± 2.2	9.8 ± 2.0	86.5 ± 34.9	3.6 ± 0.2	7.1 ± 3.1	3.5 ± 0.4	1/6	0.8 ± 0.3	31.6 ± 8.0			3.4 ± 0.3	658.8 ± 283.9	no
Statin use (n = 11)	0.55	77.5 ± 4.2	8.8 ± 1.1	64.7 ± 20.8	2.9 ± 0.5	18.3 ± 9.2	3.8 ± 0.5	2/7	1.0 ± 0.4	46.0 ± 13.0			2.2 ± 0.2	83.2 ± 53.5	yes
		⁎		⁎						⁎			⁎⁎⁎	⁎	

NA = not available.

a Time since diagnosis.

b Rai stage 0 = lymphocytosis, 1 = with adenopathy, 2 = with hepatosplenomegaly, 3 = with anemia, 4 = with thrombocytopenia.

c Normal range:0.6–23 μg/ml.

d Number of treatments including alkylator and fludarabine regimens, splenectomy, and Ibrutinib.

e Lymphocyte doubling time in months.

f Difference between densitometric values for pSTAT3 normalized to beta-actin in activated CLL cells with and without LDL supplementation (0.5 mM) after 18 h.

g Difference between HMGCoA-reductase mRNA transcripts in the presence or absence of LDLs after 18 h.

h Ratio of males to females.

i Number of intermediate/high-risk cytogenetic lesions: deletion 17p, deletion 11q, trisomy 12 (T12).

j Average ± standard error.

⁎ p < 0.05.

⁎⁎ p < 0.001.

⁎⁎⁎ p < 0.005.
