# Supplementary material for: Low Density Lipoproteins Amplify Cytokine-signaling in Chronic Lymphocytic Leukemia Cells
Source: eBioMedicine. 2016 Nov 30;15:24–35. doi: 10.1016/j.ebiom.2016.11.033 (PMC5233814; doi:10.1016/j.ebiom.2016.11.033)
Supplement: Table 1 — Patient data. [file mmc1.docx]

**Table 1: Patient Data**

| **Pt**  **No** | **Sex** | **Age**  **(yrs)** | **Time**  **(yrs)^a^** | **WBC**  **(x10^6^/ml)** | **Stage^b^** | **CD38 (%)** | **β2m^c^** | **FISH** | **Tx^d^** | **LDT**  **(mos)^e^** | **Δp-STAT3^f^** | **ΔHMGR^g^** |
| --- | --- | --- | --- | --- | --- | --- | --- | --- | --- | --- | --- | --- |
| **Gp.1** |  |  |  |  |  |  |  |  |  |  |  |  |
| 1 | f | 66 | 14 | 35 | 4 | 6 | 11.3 | 13q17p | 5 | 28 | 0.9 | -0.06 |
| 2 | f | 79 | 8 | 171 | 3 | 1 | 4.4 | 13q17p | 2 | na | 3.1 | -0.8 |
| 3 | m | 67 | 15 | 81 | 4 | 1 | 2.1 | na | 1 | na | 1.2 | -0.31 |
| 4 | m | 69 | 13 | 59 | 2 | 1 | 3.6 | na | 1 | 130 | 0.92 | -0.8 |
| 5 | f | 56 | 3 | 12 | 1 | 0 | 1.5 | na | 0 | 36 | 3.3 | -0.4 |
| 6 | m | 69 | 16 | 11 | 1 | 4 | 2.4 | na | 0 | 120 | 0.9 | -0.3 |
| 7 | m | 81 | 3 | 53 | 3 | 5 | 4 | 13q | 1 | na | 0.6 | -0.2 |
| 8 | f | 78 | 7 | 168 | 4 | 0 | 4 | normal | 2 | 29 | 0.7 | -0.4 |
| 9 | f | 78 | 11 | 55 | 4 | 12 | 3.2 | na | 0 | 18 | 1.2 | -0.13 |
| 10 | f | 53 | 9 | 81 | 1 | 1 | 2.6 | na | 0 | 30 | 1.3 | -0.03 |
| 11 | f | 66 | 11 | 26 | 4 | 17 | 7 | 13q11q | 0 | 36 | 1.5 | -0.04 |
| 12 | m | 60 | 7 | 20 | 1 | 1 | 2 | 13q | 0 | 20 | 1.2 | -0.22 |
| 13 | m | 84 | 21 | 207 | 4 | na | 4.1 | 11q | 2 | 72 | 0.8 | -0.83 |
| 14 | m | 66 | 2 | 12 | 0 | 28 | 1.8 | na | 0 | na | 2.3 | 0.06 |
| 15 | m | 62 | 7 | 71 | 3 | 2 | 2.9 | 13q | 1 | na | 1.1 | 0 |
| 16 | m | 57 | 8 | 174 | 4 | 1 | 2.7 | na | 0 | 78 | 0.7 | 0 |
| 17 | f | 75 | 4 | 27 | 4 | 5 | 3.9 | na | 0 | 28 | 0.9 | 0.1 |
| 18 | f | 53 | 14 | 51 | 1 | 4 | 1.7 | normal | 0 | 72 | 1.1 | 0.02 |
| **Gp.2** |  |  |  |  |  |  |  |  |  |  |  |  |
| 19 | f | 70 | 19 | 532 | 3 | 2 | 6.4 | normal | 3 | 6 | 0.46 | 0.14 |
| 20 | f | 79 | 5 | 182 | 4 | 1 | 13 | normal | 2 | 5 | 0.12 | 0.21 |
| 21 | m | 63 | 5 | 59 | 1 | 1 | 2.5 | na | 0 | 14 | -0.1 | 0.35 |
| 22 | f | 73 | 10 | 242 | 4 | 1 | 4.8 | 13q | 2 | 24 | 0.58 | 0.13 |
| 23 | m | 70 | 17 | 48 | 0 | 1 | 1.5 | na | 0 | 21 | 0.01 | 0.2 |
| 24 | f | 54 | 4 | 81 | 1 | 2 | 1.8 | 13q | 0 | 10 | -0.15 | 0.17 |
| 25 | m | 56 | 2 | 79 | 2 | 2 | 2.6 | t12 | 0 | 12 | 0.26 | 0.58 |
| 26 | m | 63 | 8 | 178 | 4 | 35 | 3.9 | 13q11q | 0 | 11 | 0.11 | 0.19 |
| 27 | m | 55 | 18 | 84 | 4 | 1 | 3.7 | na | 0 | 12 | 0.32 | 0.75 |
| 28 | m | 73 | 10 | 27 | 4 | 21 | 3.4 | na | 0 | 12 | 0.24 | 0.16 |
| 29 | f | 67 | 33 | 35 | 3 | 18 | 2.4 | 13q11q | 2 | 12 | 0.04 | -0.1 |

a. Time since diagnosis

b. Rai stage 0=lymphocytosis, 1=with adenopathy, 2=with hepatosplenomegaly, 3=with anemia, 4=with thrombocytopenia

c. normal range:0.6-23 μg/ml

d. number of treatments including alkylator and fludarabine regimens, splenectomy, and Ibrutinib

e. lymphocyte doubling time in months

f. difference between densitometric values for pSTAT3 normalized to beta in activated CLL cells with and without LDL supplementation (0.5mM) after 18 h

g. difference between HMGCoA-reductase mRNA transcripts in the presence or absence of LDLs

h. ratio of males to females

i. number of high-risk cytogenetic lesions: deletion 17p, deletion 11q, trisomy 12 (T12)
