# Supplementary material for: Low Density Lipoproteins Amplify Cytokine-signaling in Chronic Lymphocytic Leukemia Cells
Source: eBioMedicine. 2016 Nov 30;15:24–35. doi: 10.1016/j.ebiom.2016.11.033 (PMC5233814; doi:10.1016/j.ebiom.2016.11.033)
Supplement: Table 2 — Patient data for CLL cholesterol and LDL analysis. [file mmc2.docx]

**Table 2: Patient data for CLL cholesterol and LDL analysis**

|  | **Pt No** | **Sex** | **Age**  **(yrs)** | **Time (yrs)** | **WBC** | **Stage** | **CD38** | **β2m** | **FISH** | **Tx** | **LDT** | **LDL** | **Chol** | **Statin** |
| --- | --- | --- | --- | --- | --- | --- | --- | --- | --- | --- | --- | --- | --- | --- |
| **Statin** |  |  |  |  |  |  |  |  |  |  |  |  |  |  |
|  | 30 | f | 72 | 5 | 11 | 0 | 43 | 2.3 | na | 0 | 48 | 1.9 | 2 | yes |
|  | 31 | f | 81 | 12 | 103 | 3 | 1 | 4.2 | na | 0 | 60 | 3.2 | 2 | yes |
|  | 32 | m | 78 | 12 | 20 | 4 | 13 | 3.9 | normal | 4 | 7 | 2.8 | 1 | yes |
|  | 33 | f | 84 | 12 | 70 | 3 | 4 | 4.1 | 13q | 1 | 42 | 1.8 | 4 | yes |
|  | 34 | m | 46 | 5 | 48 | 4 | 4 | 1.9 | 13q | 1 | 18 | 1.8 | 3 | yes |
|  | 35 | f | 74 | 7 | 15 | 0 | 91 | 2.9 | na | 0 | 16 | 1.5 | 101 | yes |
|  | 36 | m | 87 | 12 | 14 | 4 | 1 | 3.4 | na | 0 | 120 | 2.5 | 100 | yes |
|  | 37 | m | 86 | 14 | 18 | 3 | na | 2.9 | 13q | 2 | 132 | 2.2 | 600 | yes |
|  | 38 | f | 91 | 5 | 236 | 3 | na | 7.8 | 11q | 1 | 28 | 2.3 | 85 | yes |
|  | 39 | m | 60 | 7 | 127 | 4 | 1 | 3.1 | 13q | 2 | 22 | 1.9 | 15 | yes |
|  | 40 | m | 93 | 6 | 50 | 4 | 7 | 5.8 | 11q | 0 | 13 | 1.9 | 2.5 | yes |
| **Group A** |  |  |  |  |  |  |  |  |  |  |  |  |  |  |
|  | 41 | m | 87 | 4 | 56 | 4 | 1 | 11.1 | 17p | 2 | 6 | 1.5 | 1.5 | no |
|  | 42 | f | 41 | 11 | 373 | 4 | 8 | 2.2 | 13q | 1 | 84 | 1.8 | 2 | no |
|  | 43 | f | 71 | 7 | 68 | 3 | 1 | 3.9 | 13q | 1 | 24 | 2.6 | 2 | no |
|  | 44 | m | 68 | 10 | 330 | 4 | 1 | 3.3 | 13q | 3 | 7 | 1.8 | 10 | no |
|  | 45 | m | 70 | 16 | 70 | 4 | 4 | 2.1 | na | 0 | 72 | 1.7 | 15 | no |
|  | 46 | f | 76 | 12 | 90 | 3 | 1 | 5.5 | 13q | 3 | 8 | 1.5 | 20 | no |
|  | 47 | m | 79 | 11 | 224 | 4 | 25 | 11.5 | t12 | 1 | 33 | 1.9 | 19 | no |
|  | 48 | m | 91 | 20 | 98 | 4 | 13 | 3.5 | 13q11q | 1 | 20 | 2.1 | 1 | no |
|  | 24 | f | 54 | 4 | 81 | 1 | 2 | 1.8 | 13q | 0 | 10 | 3.6 | 15 | no |
| **Group B** |  |  |  |  |  |  |  |  |  |  |  |  |  |  |
|  | 49 | m | 69 | 12 | 19 | 4 | 1 | 4.7 | 13q | 0 | 36 | 4.1 | 45 | no |
|  | 50 | m | 67 | 8 | 19 | 3 | 12 | 5.1 | 13q11q | 2 | 13 | 3.3 | 62 | no |
|  | 51 | m | 59 | 4 | 50 | 4 | 25 | 2.6 | na | 0 | 30 | 4.2 | 21 | no |
|  | 52 | m | 68 | 13 | 70 | 4 | 1 | 4.8 | normal | 2 | 18 | 3.1 | 100 | no |
|  | 53 | m | 68 | 5 | 73 | 3 | 1 | 4.1 | 13q | 2 | 13 | 2.9 | 21 | no |
|  | 54 | m | 73 | 10 | 15 | 4 | 21 | 2.7 | na | 0 | 22 | 2.7 | 1700 | no |
|  | 55 | m | 63 | 11 | 376 | 4 | na | 2.4 | na | 0 | 42 | 2.2 | 1500 | no |
|  | 56 | m | 55 | 3 | 15 | 2 | 1 | 1.8 | na | 0 | 14 | 4.6 | 2500 | no |
|  | 57 | f | 60 | 25 | 60 | 4 | 2 | 2.6 | 13q | 0 | 99 | 2.3 | 600 | no |
|  | 8 | f | 78 | 7 | 168 | 4 | 0 | 4 | normal | 2 | 29 | 4.3 | 39 | no |
