# Supplementary material for: Low Density Lipoproteins Amplify Cytokine-signaling in Chronic Lymphocytic Leukemia Cells
Source: eBioMedicine. 2016 Nov 30;15:24–35. doi: 10.1016/j.ebiom.2016.11.033 (PMC5233814; doi:10.1016/j.ebiom.2016.11.033)
Supplement: Supplementary file 3 — Supplementary figures [file mmc3.pptx]

## Slide 1
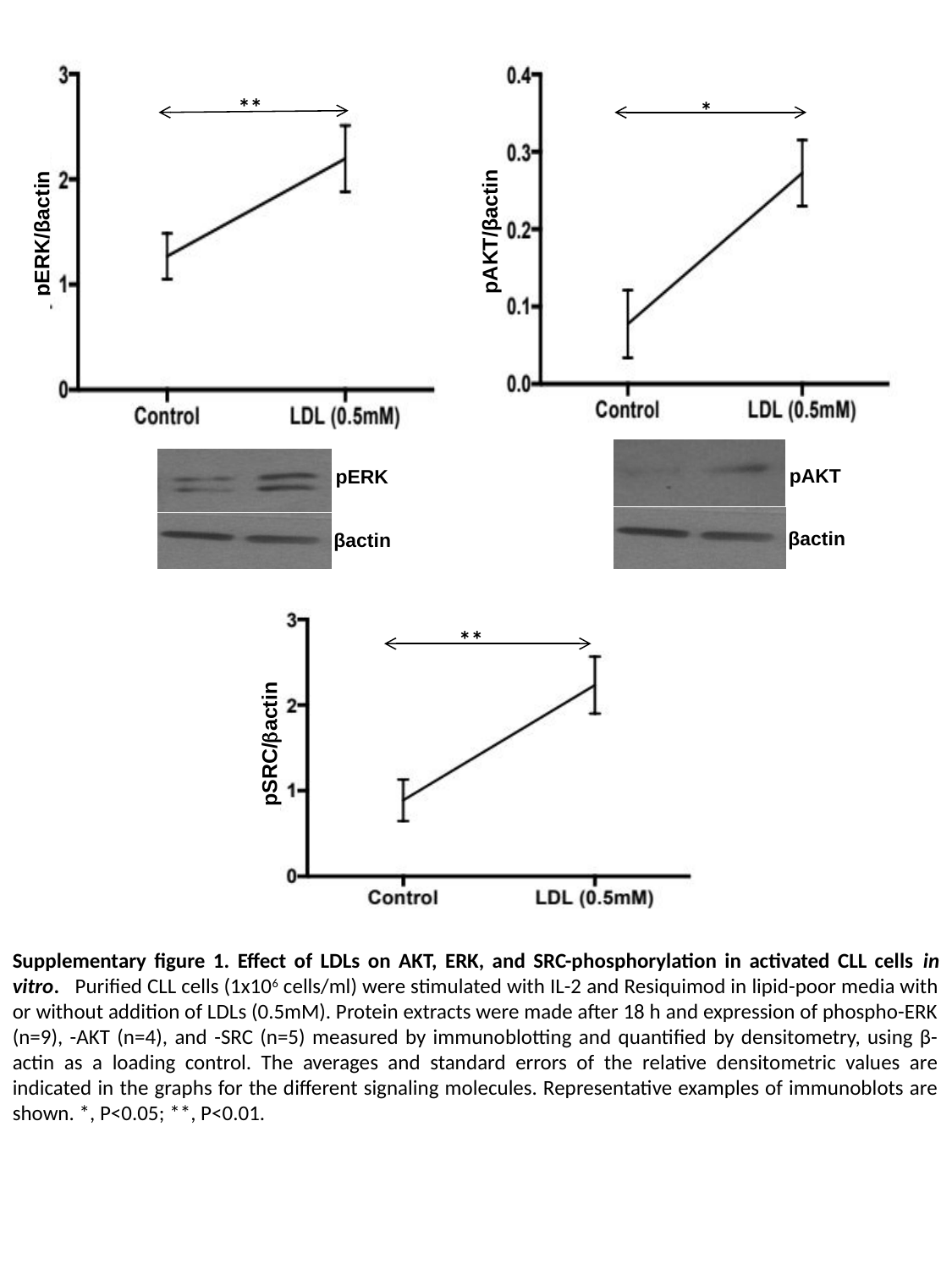

**
*
pAKT/βactin
pERK/βactin
pAKT
pERK
βactin
βactin
**
pSRC/βactin
Supplementary figure 1. Effect of LDLs on AKT, ERK, and SRC-phosphorylation in activated CLL cells in vitro. Purified CLL cells (1x10^6^ cells/ml) were stimulated with IL-2 and Resiquimod in lipid-poor media with or without addition of LDLs (0.5mM). Protein extracts were made after 18 h and expression of phospho-ERK (n=9), -AKT (n=4), and -SRC (n=5) measured by immunoblotting and quantified by densitometry, using β-actin as a loading control. The averages and standard errors of the relative densitometric values are indicated in the graphs for the different signaling molecules. Representative examples of immunoblots are shown. *, P<0.05; **, P<0.01.

## Slide 2
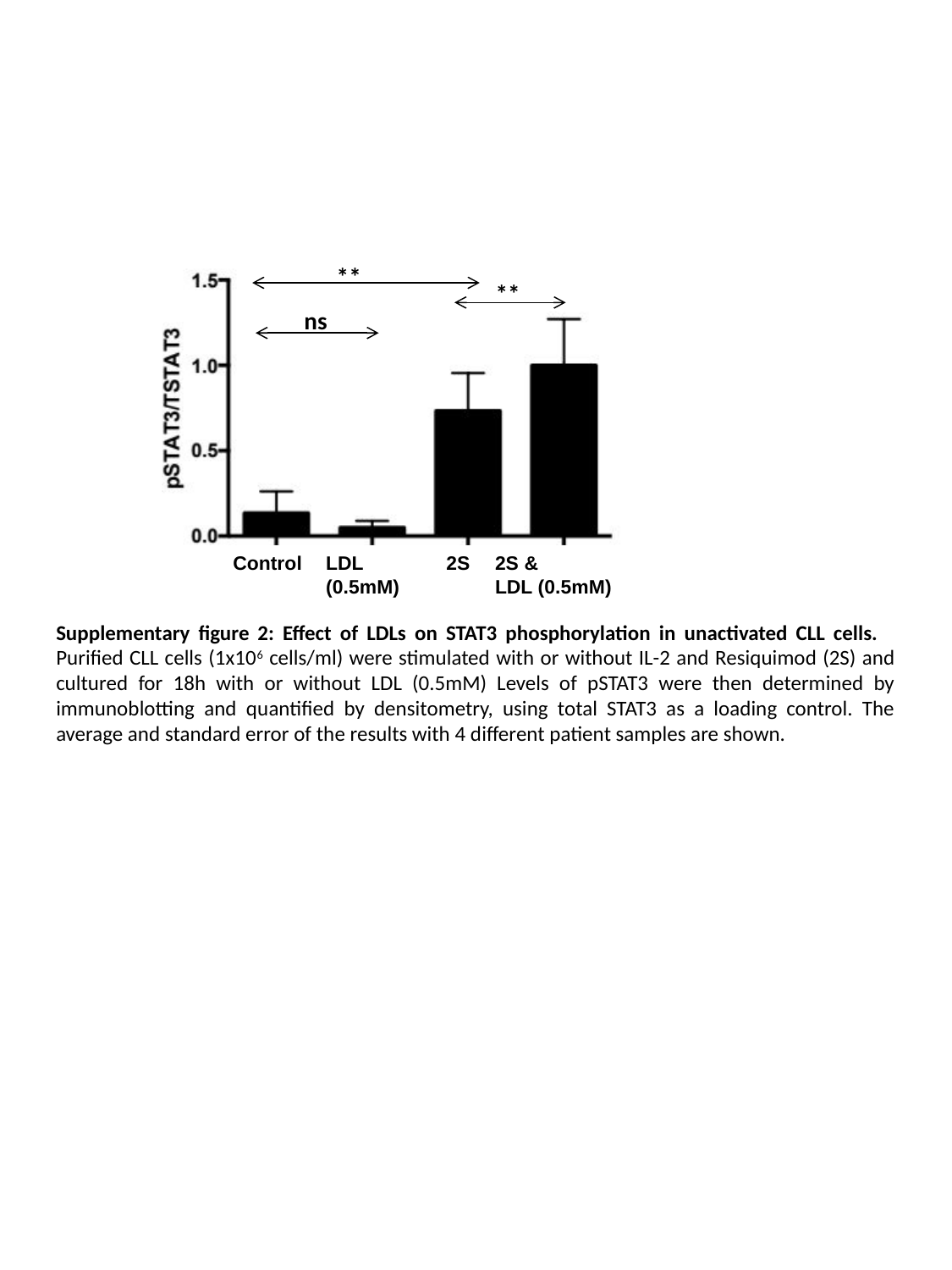

**
**
ns
Control
LDL
(0.5mM)
2S
2S &
LDL (0.5mM)
Supplementary figure 2: Effect of LDLs on STAT3 phosphorylation in unactivated CLL cells. Purified CLL cells (1x10^6^ cells/ml) were stimulated with or without IL-2 and Resiquimod (2S) and cultured for 18h with or without LDL (0.5mM) Levels of pSTAT3 were then determined by immunoblotting and quantified by densitometry, using total STAT3 as a loading control. The average and standard error of the results with 4 different patient samples are shown.

## Slide 3
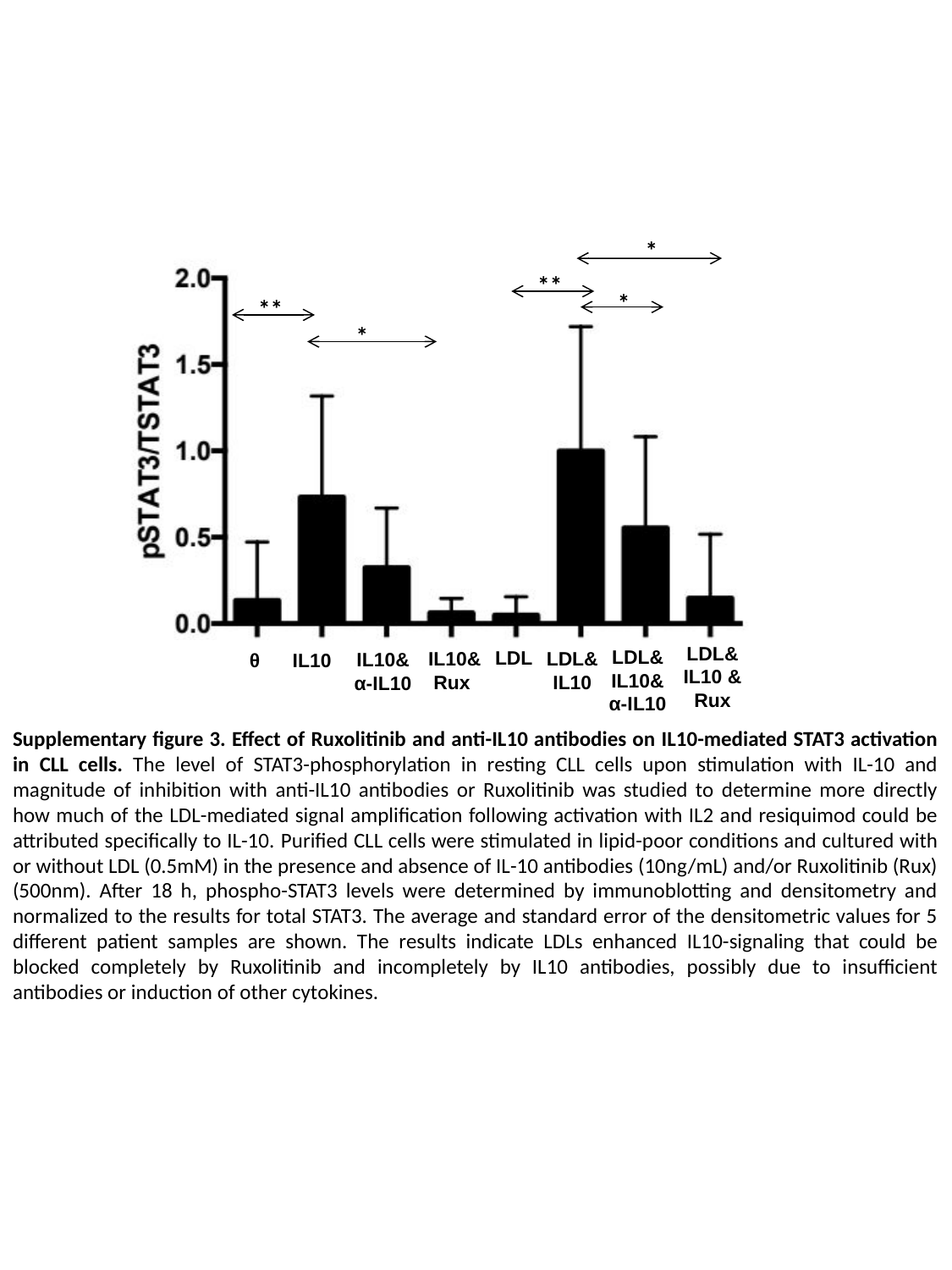

*
**
 *
**
*
LDL&
IL10 &
Rux
LDL&
IL10&
α-IL10
LDL
IL10&
Rux
LDL&
IL10
IL10&
α-IL10
θ
IL10
Supplementary figure 3. Effect of Ruxolitinib and anti-IL10 antibodies on IL10-mediated STAT3 activation in CLL cells. The level of STAT3-phosphorylation in resting CLL cells upon stimulation with IL-10 and magnitude of inhibition with anti-IL10 antibodies or Ruxolitinib was studied to determine more directly how much of the LDL-mediated signal amplification following activation with IL2 and resiquimod could be attributed specifically to IL-10. Purified CLL cells were stimulated in lipid-poor conditions and cultured with or without LDL (0.5mM) in the presence and absence of IL-10 antibodies (10ng/mL) and/or Ruxolitinib (Rux) (500nm). After 18 h, phospho-STAT3 levels were determined by immunoblotting and densitometry and normalized to the results for total STAT3. The average and standard error of the densitometric values for 5 different patient samples are shown. The results indicate LDLs enhanced IL10-signaling that could be blocked completely by Ruxolitinib and incompletely by IL10 antibodies, possibly due to insufficient antibodies or induction of other cytokines.

## Slide 4
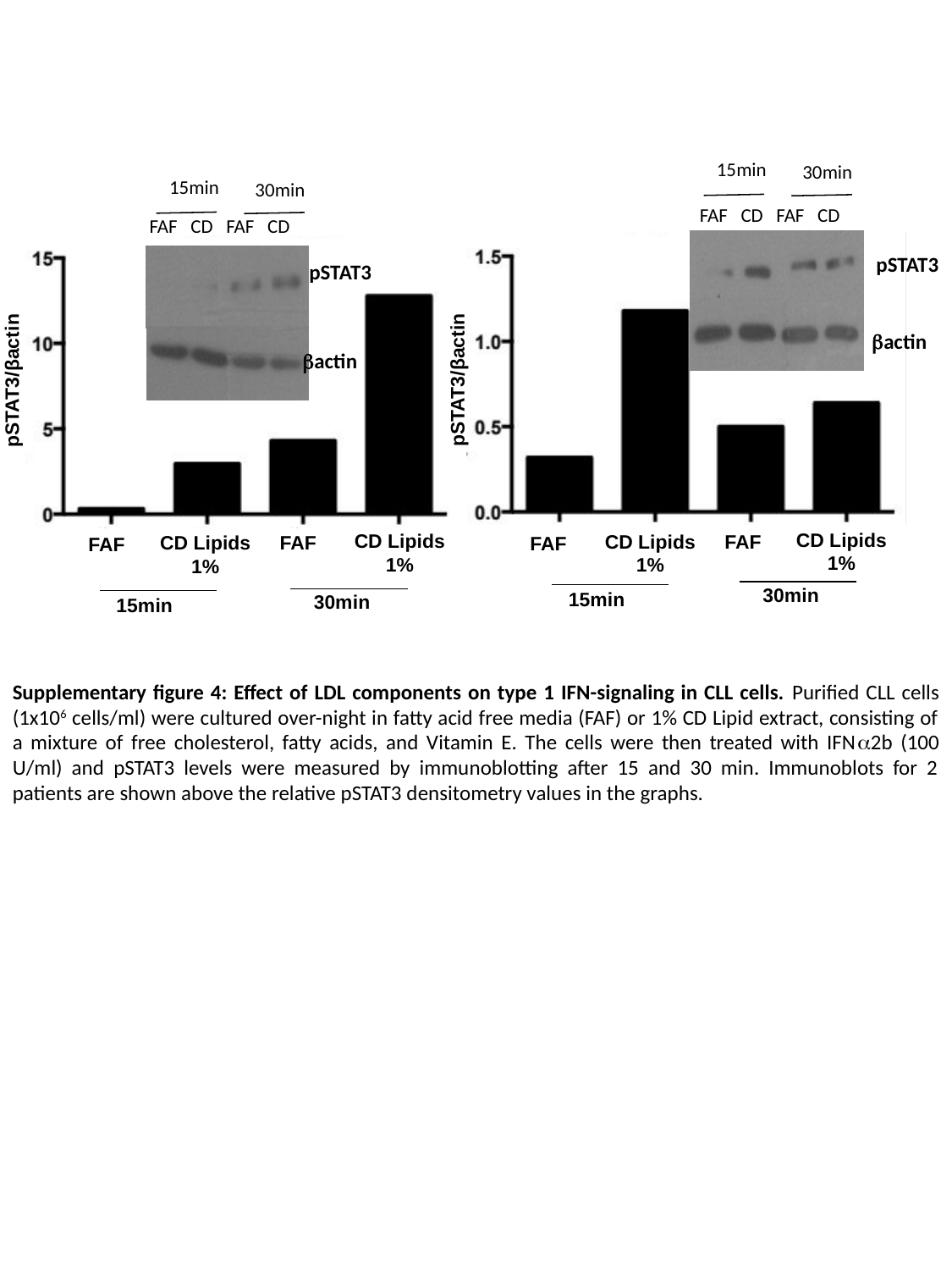

15min
30min
15min
30min
FAF CD FAF CD
pSTAT3/βactin
30min
15min
CD Lipids
1%
CD Lipids
1%
FAF
FAF
FAF CD FAF CD
pSTAT3
pSTAT3
βactin
βactin
pSTAT3/βactin
CD Lipids
1%
CD Lipids
1%
FAF
FAF
30min
15min
Supplementary figure 4: Effect of LDL components on type 1 IFN-signaling in CLL cells. Purified CLL cells (1x10^6^ cells/ml) were cultured over-night in fatty acid free media (FAF) or 1% CD Lipid extract, consisting of a mixture of free cholesterol, fatty acids, and Vitamin E. The cells were then treated with IFNα2b (100 U/ml) and pSTAT3 levels were measured by immunoblotting after 15 and 30 min. Immunoblots for 2 patients are shown above the relative pSTAT3 densitometry values in the graphs.

## Slide 5
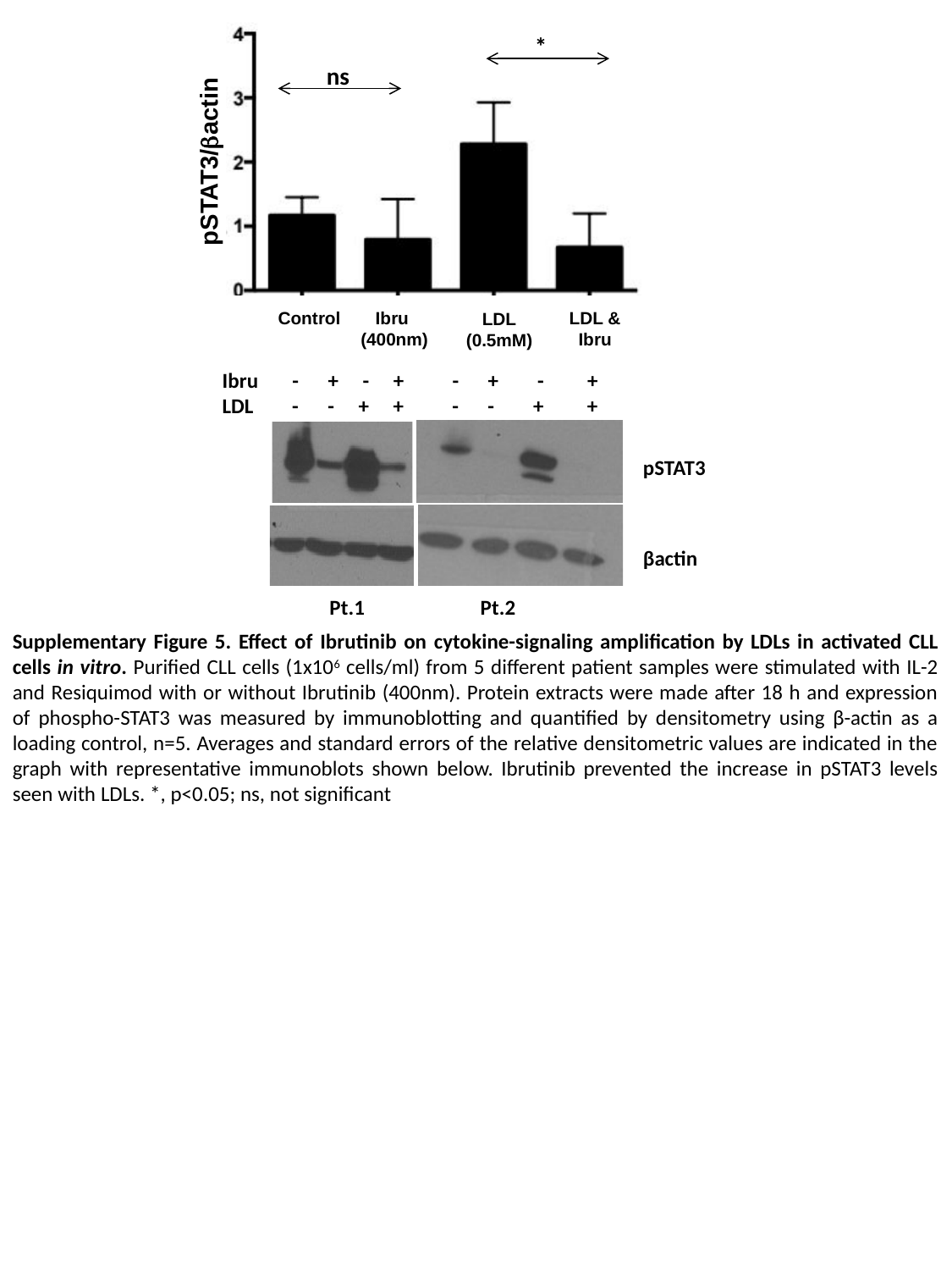

Control
Ibru
(400nm)
LDL &
Ibru
LDL
(0.5mM)
*
ns
pSTAT3/βactin
Ibru - + - + - + - +
LDL - - + + - - + +
pSTAT3
βactin
Pt.2
Pt.1
Supplementary Figure 5. Effect of Ibrutinib on cytokine-signaling amplification by LDLs in activated CLL cells in vitro. Purified CLL cells (1x10^6^ cells/ml) from 5 different patient samples were stimulated with IL-2 and Resiquimod with or without Ibrutinib (400nm). Protein extracts were made after 18 h and expression of phospho-STAT3 was measured by immunoblotting and quantified by densitometry using β-actin as a loading control, n=5. Averages and standard errors of the relative densitometric values are indicated in the graph with representative immunoblots shown below. Ibrutinib prevented the increase in pSTAT3 levels seen with LDLs. *, p<0.05; ns, not significant

## Slide 6
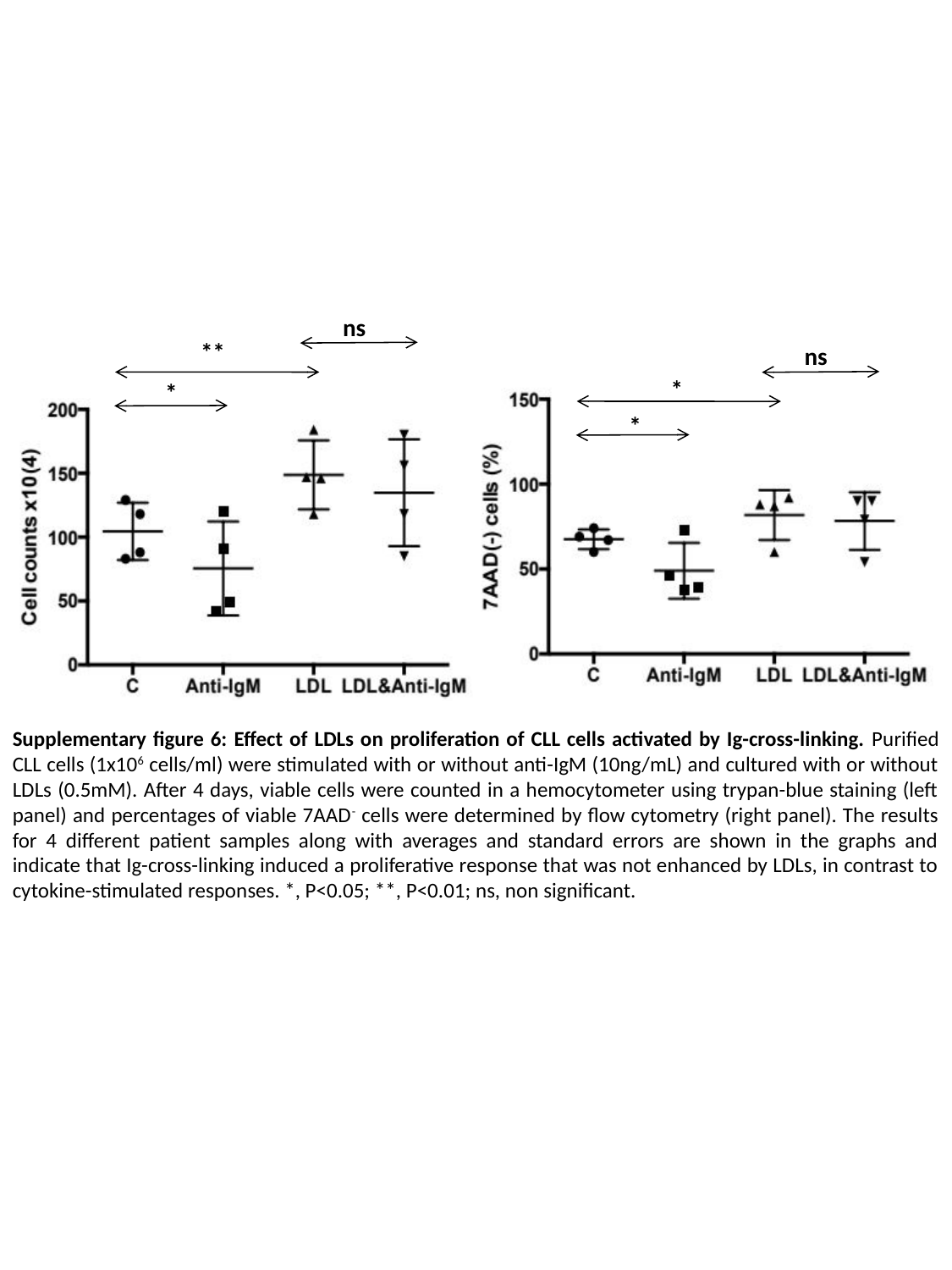

ns
**
*
ns
*
*
Supplementary figure 6: Effect of LDLs on proliferation of CLL cells activated by Ig-cross-linking. Purified CLL cells (1x10^6^ cells/ml) were stimulated with or without anti-IgM (10ng/mL) and cultured with or without LDLs (0.5mM). After 4 days, viable cells were counted in a hemocytometer using trypan-blue staining (left panel) and percentages of viable 7AAD- cells were determined by flow cytometry (right panel). The results for 4 different patient samples along with averages and standard errors are shown in the graphs and indicate that Ig-cross-linking induced a proliferative response that was not enhanced by LDLs, in contrast to cytokine-stimulated responses. *, P<0.05; **, P<0.01; ns, non significant.

## Slide 7
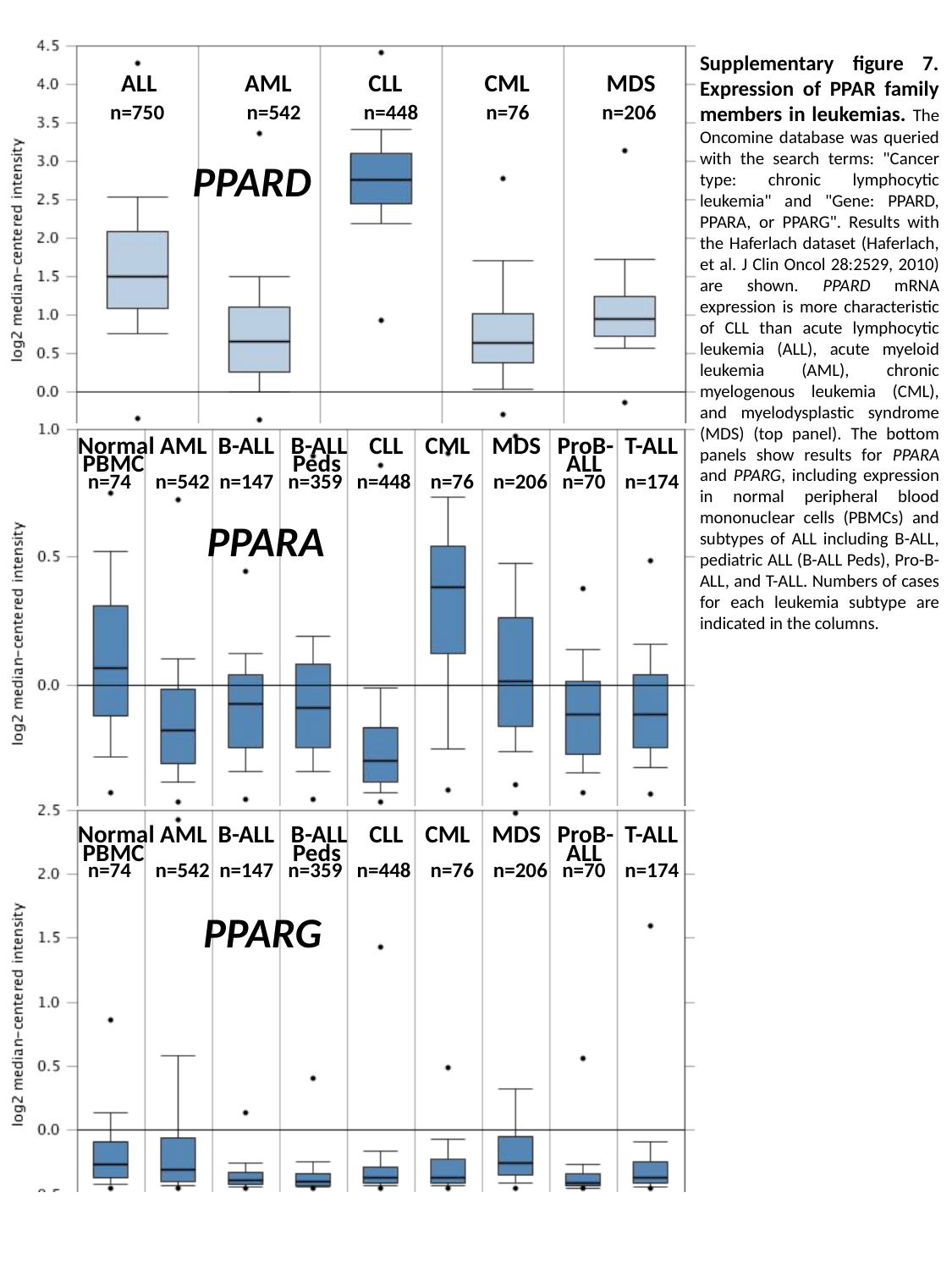

ALL AML CLL CML MDS
n=750 n=542 n=448 n=76 n=206
Normal AML B-ALL B-ALL CLL CML MDS ProB- T-ALL
PBMC Peds ALL
n=74 n=542 n=147 n=359 n=448 n=76 n=206 n=70 n=174
Normal AML B-ALL B-ALL CLL CML MDS ProB- T-ALL
PBMC Peds ALL
n=74 n=542 n=147 n=359 n=448 n=76 n=206 n=70 n=174
PPARD
PPARA
PPARG
Supplementary figure 7. Expression of PPAR family members in leukemias. The Oncomine database was queried with the search terms: "Cancer type: chronic lymphocytic leukemia" and "Gene: PPARD, PPARA, or PPARG". Results with the Haferlach dataset (Haferlach, et al. J Clin Oncol 28:2529, 2010) are shown. PPARD mRNA expression is more characteristic of CLL than acute lymphocytic leukemia (ALL), acute myeloid leukemia (AML), chronic myelogenous leukemia (CML), and myelodysplastic syndrome (MDS) (top panel). The bottom panels show results for PPARA and PPARG, including expression in normal peripheral blood mononuclear cells (PBMCs) and subtypes of ALL including B-ALL, pediatric ALL (B-ALL Peds), Pro-B-ALL, and T-ALL. Numbers of cases for each leukemia subtype are indicated in the columns.
